# Energy-Based Methods and Nanocarrier-Based Approaches for Melasma Treatment

**DOI:** 10.34172/apb.42794

**Published:** 2024-09-15

**Authors:** Amiremad Kheirieh, Amirhessam Kheirieh, Zahra Mahdavi, Ali Mohammad Halvani, Amir Mohammad Bagheri, Hooriyeh Nassirli, Shiva Golmohammadzadeh, Bizhan Malaekeh-Nikouei

**Affiliations:** ^1^Department of Pharmaceutics, School of Pharmacy, Mashhad University of Medical Sciences, Mashhad, Iran.; ^2^Clinical Research Development Unit, Bahar Hospital, Shahroud University of Medical Sciences, Shahroud, Iran.; ^3^Student Research Committee, School of Pharmacy, Mashhad University of Medical Sciences, Mashhad, Iran.; ^4^Pharmaceutics Research Center, Institute of Neuropharmacology, Kerman University of Medical Sciences, Kerman, Iran.; ^5^Pharmaceutical Research Center, Pharmaceutical Technology Institute, Mashhad University of Medical Sciences, Mashhad, Iran.; ^6^Nanotechnology Research Center, Pharmaceutical Technology Institute, Mashhad University of Medical Sciences, Mashhad, Iran.

**Keywords:** Melasma, Hyperpigmentation, Energy-based methods, Nanocarriers

## Abstract

**Purpose:**

Melasma is a persistent skin condition caused by excessive melanin production, particularly affecting women’s quality of life. It can result from various factors like sun exposure, genetics, hormones, medications, or inflammation. Effective melasma treatment requires products that can deeply penetrate the skin. The outermost skin layer, known as the stratum corneum (SC), plays a crucial role in delivering topical and transdermal drugs. Researchers have developed numerous strategies to enhance skin permeability and drug efficacy.

**Methods:**

This review delves into energy-based techniques and nanocarrier systems for treating melasma, specifically focusing on improving drug delivery to the viable epidermis (EP) while overcoming the SC barrier.

**Results:**

Physical methods offer benefits such as enhanced skin penetration but come with drawbacks like frequent visits, high costs, and the need for specialized equipment and skilled operators. Microneedle patches are gaining attention as a convenient physical treatment option for delivering multiple medications effectively, offering targeted delivery and minimal side effects. Nanocarrier systems like transferosomes demonstrate promise in enhancing skin penetration for treating melasma and skin hyperpigmentation. While they offer advantages such as high drug entrapment and improved bioavailability, challenges like stability issues and scalability hinder their widespread adoption.

**Conclusion:**

Energy-based techniques enhance drug penetration but can lead to scarring and burns, while dissolvable micro-needles offer a convenient and effective alternative. Nano-drug carriers, like nanostructured lipid carriers (NLCs) and transferosomes, show promise for improved skin drug delivery with their flexible structures and enhanced penetration capabilities, yet further clinical research is needed for definitive conclusions

## Introduction

**Figure 1:**
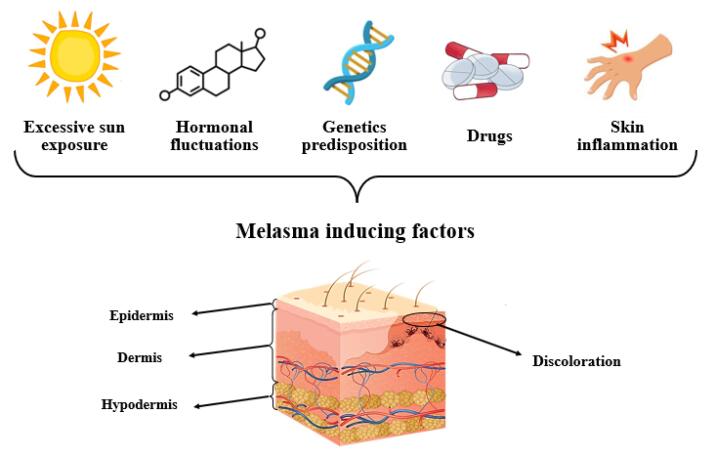
Factors inducing melasma: Excessive sun exposure, hormonal fluctuations, genetic predisposition, drugs, and skin inflammation

 Melasma is an unusual pigmentary skin condition that occurs as symmetrical hyperpigmented skin with uneven borders caused by multiple factors, including external exposure to solar radiation, hormonal changes, skin inflammation, and genetic predisposition (Figure 1). The skin can experience focal hypermelanogenic phenotype due to hyper-functional melanocytes and other structural and functional abnormalities.^1^ It is more common in females and dark-skin phototypes, especially sun-exposed skin. According to the severity of the disease and hyperpigmented patches, therapeutic strategies may involve epidermal, dermal, or mixed approaches. Besides, patients ‘compliances and skin type, treatment adherence, former responses to treatment, financial concerns, and strict restrictions on sun exposure are among the essential considerations in choosing the appropriate treatment strategies. Melasma requires prolonged therapy and is treated with various chemical and physical methods. Topical lightening agents are the first line of treatment. Nowadays, physical energy-based methods such as micro-needling (MN) or radiofrequency (RF) are drawing scientists’ interest. Despite, monotherapy with these devices can cause rebound lesions and have limitations. On the other hand, combination of physical methods with nanocarriers such as microemulsions (MEs), nanoemulsions (NEs), lipid nanoparticles (LNPs), solid lipid nanoparticles (SLNs), and nanostructured lipid carriers (NLCs) can enhance drug delivery with lower side effects. Vesicular nanocarriers like transferosomes and ethosomes are also effective in deep skin penetration. Natural polymeric nanoparticles like chitosan are excellent for targeted drug delivery to skin organelles.^2^

 Although numerous therapeutic strategies such as topical lightening creams (hydroquinone (HQ), arbutin, kojic acid, vitamin C, and azelaic acid) and energy-based approaches (laser, MN, iontophoresis, etc.) have been introduced for skin drug delivery now, they have not shown satisfactory outcomes. In this context, the ability of active pharmaceutical ingredients to effectively penetrate melanocytes and suppress their excessive activity is critically impaired due to the presence of skin barriers.

 The outermost layer of skin is called the stratum corneum (SC). It comprises dead, flattened, and enucleated corneocytes encased in a lipid extracellular matrix.^3^ The viable epidermis (EP) is located below SC and primarily comprises keratinocytes, containing about 40% protein, 40% water, and 15%–25% lipids, creating a hydrophilic environment.^4^ Since melanocytes are mainly found in the basal layer of EP, therapeutics delivery into the viable EP is critical for the topical treatment of melisma.^5^ It should be noted that the lipophilic environment of SC facilitates the ability of highly lipophilic molecules to penetrate, aggregation and stay in this layer. Therefore, only uncharged molecules with mild lipophilicity can cross the SC and pass into the viable EP. While highly lipophilic molecules are prone to remain in the SC and cannot readily penetrate the viable EP.^6^ In this paper, we have reviewed energy-based and novel delivery methods like nanocarriers to enhance the efficacy of drug delivery to EP and passage through SC for the treatment of melisma.

## Methods

 A comprehensive review was conducted on energy-based approaches (micro-needle patches, MN, iontophoresis, sonophoresis, laser, and RF), nanoparticles (MEs, NEs, SLNs, NLCs, liposomes, niosomes, ethosomes, invasomes), and polymeric nanoparticles (chitosan-based nanoparticles) that enhanced drug delivery in melasma treatment. We used PubMed, Scopus, Google Scholar, and Web of Science search engines and databases from 1990 to 2023. Articles were further selected based on their type of study, and we included clinical trials, animal trials and *in-vitro* studies in our review. From clinical trials, we summarized statistically significant results such as year, sample size, medication assessed, treatment duration, treatment follow-up, and MASI or mMASI. Other relevant or animal research was evaluated to find any information related to melasma treatment. Then, we compared MASI or mMASI of clinical trials of different methods to identify which physical method or nanoparticles has a better response in enhancing drug delivery for melasma treatment. Finally, information was sorted and discussed based on physical methods and nanocarriers to enhance skin penetration and melasma treatment.

**Figure 2:**
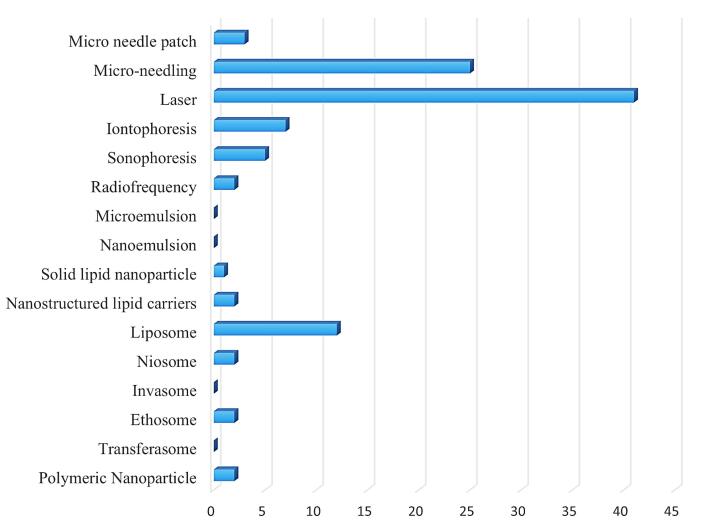
Clinical trials on melasma drug delivery from 2000 to 2023

 In the literature search, we found a total of 1659 articles. Among these, 144 were duplicates. From the remaining articles, we selected 103 clinical trials on micro-needle patches (n = 3), MN(n = 25), iontophoresis (n = 7), sonophoresis (n = 5), laser (n = 41), RF(n = 2), liposome (n = 11), ethosome (n = 2), noisome (n = 2), SLNs (n = 1), NLCs (n = 2) and polymeric nanoparticle (n = 2). Then we assessed *in-vitro* and animal studies to get further information on other subjects. We reported and compared between studies with following terms: MASI, mMASI, aMASI, Hemi-MASI, MOPASI, Luminance value (L value), and MI (Figure 2).

## Quantitative assessment of melasma

**Figure 3:**
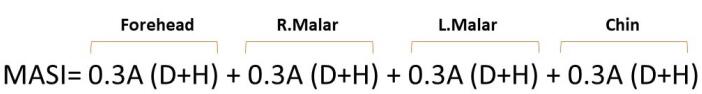
Calculation of Melasma Area and Severity Inde

 In 1994, Kimbrough Green et al^7^ introduced the Melasma Area and Severity Index (MASI) as the first scoring system aimed to provide a more accurate criterion for quantitative assessment of melasma severity and qualitative changes during the treatment (Figure 3). Its purpose was to establish a measurable criterion for evaluating melasma response in clinical trials, taking into account the affected area and characteristics such as darkness and homogeneity of skin.^7^ MASI is based on a scoring system similar to the one developed for psoriasis, which considers the extent and degree of pigmentary alterations. As it said, it incorporates three components: area, darkness, and homogeneity. These components are measured in four facial areas (A): the forehead (30%), right malar region (30%), left malar region (30%), and chin (10%). Each area is assigned a score ranging from 0 to 6, indicating the percentage of involvement (0 = no involvement; 1 = ≤ 10%; 2 = 10-29%; 3 = 30-49%; 4 = 50-69%; 5 = 70-89%; and 6 = 90-100%). The darkness (D) component assesses the melasma pigmentation relative to normal skin, and it is graded from 0 to 4 (0 = normal skin color; 1 = barely visible hyperpigmentation; 2 = mild hyperpigmentation; 3 = moderate hyperpigmentation; and 4 = severe hyperpigmentation). Similarly, the homogeneity (H) component evaluates the uniformity of hyperpigmentation and is scored from 0 to 4 (0 = normal skin color without hyperpigmentation; 1 = specks of involvement; 2 = involvement with spot size less than 1.5 cm in diameter; 3 = involvement with spot size greater than 2 cm in diameter; and 4 = involvement with uniform hyperpigmentation without any healthy skin). The MASI score is calculated by summing the darkness and homogeneity scores, multiplied by the percentage area involved and its severity. The total MASI score ranges from 0 to 48, with 48 representing the most severe melisma.^8^

 However, Pandya et al^9^ in 2011, who was evaluating the validity of the MASI, found challenges associated with its use in clinical settings and disease diagnosis. They argued that the subjectivity of the MASI leads to variability among different evaluators, even after prior training, and it is also time-consuming. They also concluded that the removal of the homogeneity component does not affect the validity of the index. Consequently, the authors proposed a modified version of MASI, known as modified MASI (mMASI), which simplifies the calculation by removing the homogeneity component. However, mMASI does not address the issues of inter-rater scoring variability and the need for training to increase scoring reliability that are present in the original MASI.^8^

 Calculation of modified melasma Area and severity index:


$$\begin{array}{l} Modified\;MASI\;total\;score=0.3A\left(forehead\right)*D\left(forehead\right)\\ +0.3A\left(left\;malar\right)*D\left(left\;malar\right)+0.3A\left(right\;malar\right)\\ {}*D\left(right\;malar\right)+0.1A\left(chin\right)*D\left(chin\right) \end{array}$$

Additionally, a computer-based approach called automated MASI (aMASI) was introduced as a pilot study by Tay et al^10^ in 2015. While it offers advantages such as increased accuracy and speed in scoring, it has limitations, including difficulty in distinguishing melasma from other hyperpigmentation disorders and the requirement of standardized lighting and fixed angles for imaging. aMASI algorithm still requires further validation and optimization in future studies. To summarize, MASI and mMASI are the gold standards for diagnosing melasma. These scoring systems are commonly used in clinical trials to assess the efficacy of melasma treatments. Introducing aMASI system, utilizing computer image analysis, shows promise but still requires further validation and optimization.

 Hemifacial MASI (Hemi-MASI) is a specialized scoring system used to assess the severity of melasma on one side (hemifacial) of the face. The Hemi-MASI scoring system typically involves dividing the face into different regions and assigning scores based on the extent and intensity of melasma in each area. The scores for each part are then added together to obtain a total Hemi-MASI score. A higher score indicates more severe melisma.^11,12^

 Mottled Pigmentation Area and Severity Index (MoPASI), is an assessment tool created from the MASI scale used to evaluate pigmentation in four face areas. Each area is assigned a different weight, as follows: forehead (0.2), nose, upper lip, chin (0.2), left cheek, periorbital region (0.3), right cheek, and periorbital region (0.3). Three variables are evaluated in each area: percentage of extent involved with pigmentation (A), darkness of pigment in the area (D), and pattern of pigment (P).^13^ The luminance value represents the brightness or intensity of light emitted or reflected from an object. It is often expressed numerically on a scale from 0 to 100, where 0 represents absolute darkness (black), and 100 represents the maximum brightness (white). By comparing the luminance values of hyperpigmented areas against adjacent non-pigmented or normal skin, researchers might seek to assess the contrast or relative difference in brightness levels. This analysis could provide insights into the severity or extent of hyperpigmentation, particularly concerning the surrounding skin.^14^

 The Melanin Index (MI) is the melanin level to total pigments in a skin sample. So, the MIis a parameter mainly influenced by melanin content. It serves as an objective measure of skin color.^15-17^

## Physical energy-based methods for enhancing transdermal and topical delivery

**Figure 4:**
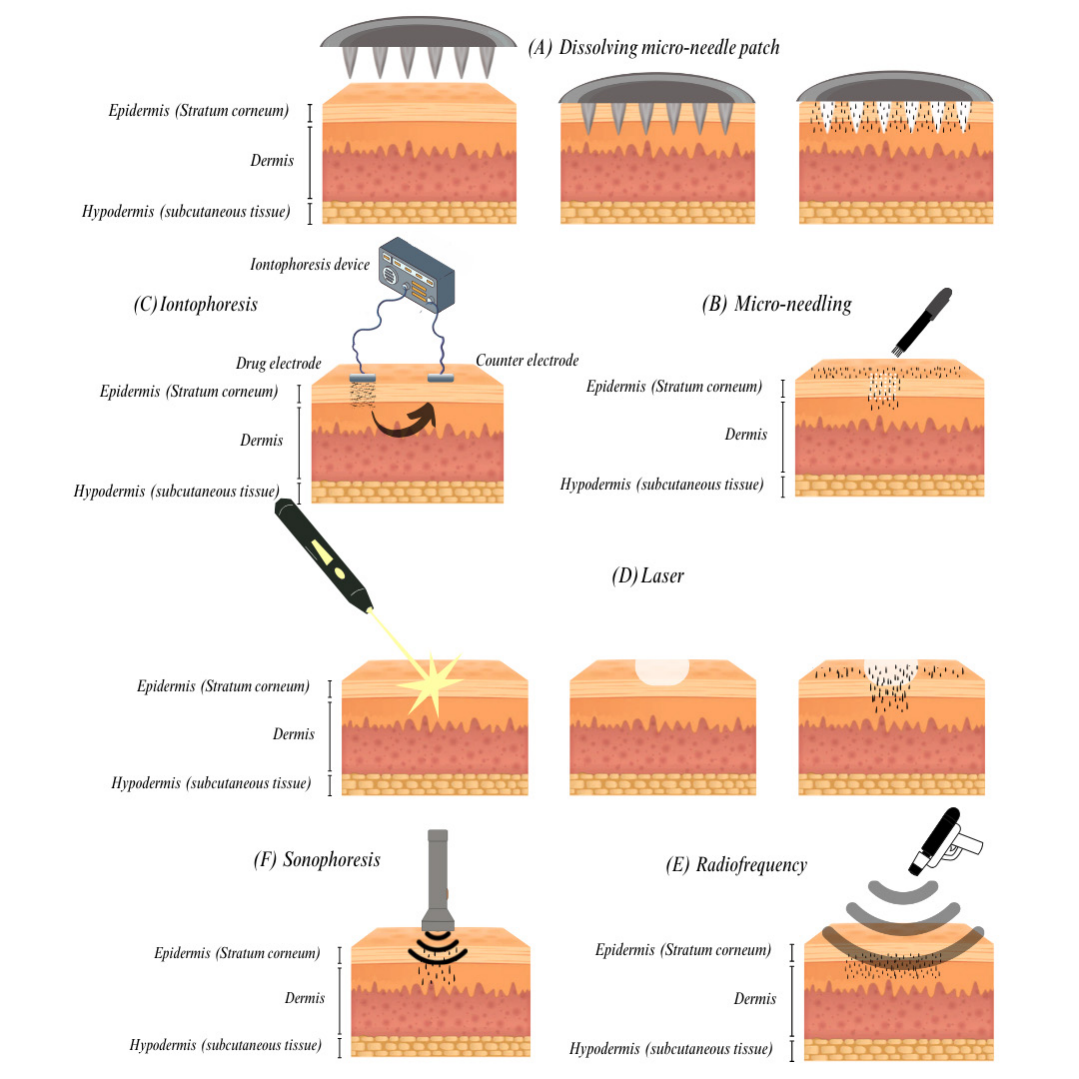
Energy-based methods; Micro-needle patches (A), Micro-needling (B), Iontophoresis (C), Laser (D), Radiofrequency (E), and Sonophoresis (F)

 Physical penetration approaches for transdermal and topical delivery have advanced with the rapid development of microelectromechanical systems and artificial intelligence. In this context, several energy-based methods have recently been introduced, including micro-needle patches, MN, iontophoresis, sonophoresis, RF, and lasers (Figure 4). These methods can directly alter the surface structure of the skin to improve transdermal absorption. Applying an energy source to the skin’s surface can increase the solutes’ diffusivity through the SC to reach deeper layers.^18^ Recently, these technologies have been widely used due to their higher bioavailability, efficacy, and safety. They show a better choice to facilitate active pharmaceutical ingredient’s transdermal absorption than conventional methods.^19,20^

###  Micro-needle patches

 Micro-needles are an increasingly popular method for delivering cosmetic or therapeutic compounds topically or transdermally. They create microchannels by penetrating the skin’s SC layer, enabling the delivery of cosmetic and therapeutic agents.^21^ For effective skin depigmentation, the agent must be delivered to melanocytes, where melanin is synthesized.^22^ However, the SClimits the efficacy of depigmenting products.^23^ There are several types of micro-needles, solid micro-needles, hollow micro-needles, coated micro-needles, hydrogel-forming micro-needles, and dissolving micro-needle (DMN).^21^ Solid microneedles are utilized in “poke and patch” systems to create microchannels through the skin for subsequent replacement with a drug-loaded patch, solution, or cream. After removing the solid micro-needles, the skin quickly heals and closes the created microchannels.^24,25^ Hollow micro-needles have an empty cavity inside each needle and a bore on the tip, allowing drug solutions to be injected into the skin using a ‘poke and flow’ mechanism.^26^ Coated micro-needles eliminate the need for a two-step application by coating solid needles with a thin therapeutic agent layer. The coating is achieved via dip coating, ink-jet printing, or spray drying.^27^ Hydrogel micro-needles composed of cross-linked polymers increase in size upon insertion into the skin due to their hydrophilic nature and the presence of interstitial fluid. They can serve as a means to deliver drugs into or through the skin, as well as passively extract interstitial fluids, potentially allowing their use as a diagnostic tool.^28^ DMN is commonly made by encapsulating drugs in biodegradable polymers. Once the micro-needles penetrate the SC, the polymer dissolves and releases the drug.^29^

 Several clinical trial studies evaluate the effect of patches on skin hyperpigmentation.^22,23,30^ Kim et al^22^ developed a DMNpatch treating hyperpigmentation in a randomized, double-blind, placebo-controlled trial. 4-n-butylresorcinol was used as a depigmentation agent in a study involving forty-five women with skin conditions such as melasma, lentigo, freckles, and post-inflammatory hyperpigmentation. The participants were divided into two groups: one applied a 4-n-butylresorcinol patch and a control patch every three days for eight weeks, while the other group applied patches every four days for the same duration. Over the course of 8 weeks, the MI for the 3-day interval group decreased by 1.56% and 3.57% at 4 and 8 weeks, respectively, compared to the initial measurements. On the other hand, the MI for the 4-day interval group decreased by 0.94% and 1.78% at 4 and 8 weeks, respectively. The study concluded that the 3-day interval application was more effective than the 4-day interval application. The DMN patch containing 4-n-butylresorcinol was found to effectively and safely target melanocytes for skin depigmentation.

 The clinical effectiveness of dissolvable microneedles containing anti-melanogenic compounds for treating hyperpigmentation was assessed by Avcil et al.^30^ DMN with a base of hyaluronic acid (HA) were loaded with niacinamide, ascorbic acid 2-glucoside, tranexamic acid (TXA), resveratrol, 4-n-butyl-resorcinol, and *Halidrys siliquosa* extract. HA microneedle patches were created using the DAB technique, where each droplet was air-blown to solidify. Twenty patients with hyperpigmentation disorders applied test patches to the designated area for 12 weeks. The individual topology measurements showed a 51.4% improvement in the hyperpigmented zone. All individuals showed healthy skin in the test area before, during, and after the intervention, and patches were well tolerated. The study found micro-needle patches substantially improved skin color and appearance. Another study by Tai et al^23^ applied DMNto treat post-inflammatory hyperpigmentation. The efficacy of micro-needle comprised of anti-acne agents was evaluated in a 28-day clinical trial on 30 individuals. Participants applied patches once a day. Patches showed promising efficacy for improving skin pigmentation, in which skin melanin was decreased by 7.83% after 28 days of treatment without adverse skin reactions. We searched https://clinicaltrials.gov/ with the terms “melasma” and “micro-needle patch” but no other clinical trial was found.

###  Micro-needling (MN)

 As a direct penetration enhancement method, MN penetrate the skin to create permeation channels. These tiny arrays can promote transdermal penetration and drug accumulation in particular areas, providing an alternative approach to traditional hypodermic and subcutaneous injections. Recently, MN has been subject in academia and business, particularly for immunization. Moreover, their progress toward the clinic is ongoing with substantial research.^31^

 MN can pass through the SC without stimulating the local nerves and related responses.^28^ Their ability to prevent the first-pass effect and delivery of various medications even with high molecular weight (such as peptides, proteins, and DNAs), as well as the potential for regulated drug administration by integrating with a patch system, are possible advantages of MN.^31,32^ To make MN in large quantities, micro tools and microelectronics are required. Micro-needles must also be applied with sufficient force to prevent breakage or bending prior to insertion. Only micro-needles with the suitable form and necessary physical characteristics can be used efficiently into the skin.^33,34^ Several studies investigated MN to improve drug delivery and enhance treatment for melasma.

**Figure 5:**
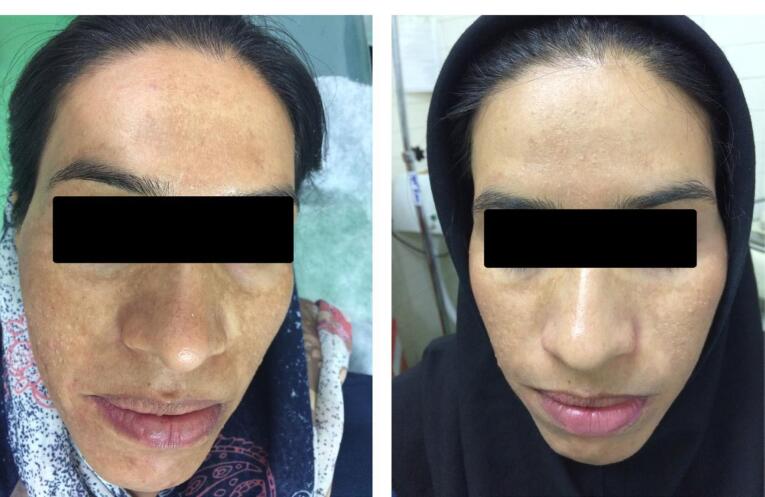
Improvement in melasma is shown in a picture: Baseline (left) and (right) after three months of treatment with microneedle and tranexamic acid. Reprinted with permission from Shamsi Meymandi et al^40^

 Lima et al^35^ studied the efficacy of Kligman’s triple-combination cream (TCC) for treating melasma with MN. Patients came to the clinic for MN (1.5 mm) on days 0 and 30 from the beginning of melasma diagnosis. They were treated with daily TCC and broad-spectrum sunscreen for 45 days. By measuring MASI standard, an average reduction of 70% was observed at the end of the treatment period. According to their results, it can be concluded that combined classical treatment (daily TCCand broad-spectrum sunscreen), along with skin MN, improved the clinical and histological characteristics of facial melasma. A study conducted by Budamakuntla et al^36^ compared the efficacy and safety of topical TXA with MNversus TXA microinjection in melasma treatment. Sixty individuals enrolled the study, who presented MASI improvement in MNgroup was 44.41% showing excellent efficacy compared to microinjection at 35.72%. Authors suggest MNas a better therapeutic option because of deeper drug delivery through microchannels created by MN. No significant side effects were reported. Also, Armit et al^37^ concluded MN as an effective way to enhance drug delivery of TXA to melasma. In a study with 40 volunteers, 65.92 improvements in mMASI score were seen in topical TXAwith MNcompared to 20.75% of a control group. However, some adverse effects were reported which was minor and well tolerated: dryness (55% of patients), persistent local erythema (17.5%), localized pruritis (12.5%), transient hyperpigmentation (5%), and localized burning (2.5%). Also, Howyda et al^38^ reported topical TXA with MNare effective for melasma treatment compared to intradermal injection of a drug. MNpresented more patient compliance. Da Silva Bergmann et al^39^ found that topical administration of retinoic acid with MNcan cause a significant improvement within two months in patients with melasma. The average MASI score among 42 subjects revealed a mean 2.839 revision compared to the control group, which was 0.8. MNof retinoic acid appears to be effective 60-day treatment for melasma. However, topical retinoic acid with MNmay reduce the non-enzymatic antioxidant defense, which is essential to protect against oxidative stress. In a trial study, Meymandi et al^40^ compared the effectiveness of 4% TXA solution with MNvs 4% HQ. Both methods did not differ in the treatment of melasma, with a noticeable improvement on both sides of the face over three months (Figure 5). Although MN can enhance trans-dermal drug absorption and improve efficacy in treating deep melasma. MN approach either with or without TXA improves skin photoaging patterns, while oral TXAinhibits the angiogenesis. In another research by Attar et al,^41^ the safety and efficacy of topical TXA versus vitamin C through MNapproach was evaluated. Using topical TXAor vitamin C after derma pen MNeffectively reduced the hemi-MASI score in melasma treatment with minimal side effects.

 Effects of MN with or without TXAwere investigated on melasma treatment by Saleh et al.^42^ Clinically, both groups showed a significant decrease in the MASI score. MNalone resulted in a lightening effect, while a combination of topical TXA with MNproduced more satisfactory results, suggesting the effectiveness of topical TXA was significantly enhanced by MN. Long-term follow-up is required to confirm the efficacy results of combined therapy in treating melasma as it is cost-effective, easily accessible, and safe with no significant adverse effects. In a study, Farag et al^43^ compared the effectiveness of topical methimazole with MNor placebo as a control. Significant improvements were observed in both clinical and dermoscopic evaluations. The methimazole-treated right sides showed a decrease in hemi-MASI. The percent of hemi-MASI score improvement was significantly associated with melasma’s malar pattern and epidermal type. No significant local or systemic side effects were observed. Methimazole can potentially be a safe and promising therapeutic agent for treating melasma. It can be used with derma pen-delivered MN.Zaky et al^44^ studied effects of topical HQ4% versus topical TXA 4% in melasma treatment. In conclusion, both treatment modalities have demonstrated safety and efficacy with minimal side effects. However, MN therapy requires frequent visits and increases the financial burden on patients, which may strongly influence patient adherence. Conversely, conventional 4% HQ cream has remained an effective and potent therapy for melasma. It has achieved higher satisfactory results among raters and subjects, although the difference was not statistically significant. More controlled and extensive sample trials are required to establish an optimal and effective treatment for melasma. In another study, Musarrat Hussain et al^44^ compared the efficacy of TXAor vitamin C with MN. TXAand vitamin C are effective adjuvant therapies for melasma when combined with MN. The mMASI significantly improved after six weeks of treatment. Another study conducted by Hofny et al,^45^ the effectiveness of trichloroacetic acid with MNversus trichloroacetic acid alone was investigated. After 1 and 3 months of treatment, all two groups showed a significant improvement in mean scores for MASI, mMASI. Combining trichloroacetic acid peel with MN is a safe and effective treatment option for melasma. Kuster Kaminski Arida et al^46^ studied the therapeutic effect of conventional TCCcombined with topical administration of TXA with MNfor melasma. In this study, 20 patients with a precise diagnosis of melasma were chosen. During the three-month treatment period, patients visited once every 30 days to receive MNand triple therapy every day. On one side of the face, a 0.9% saline solution served as a placebo, and the other half received a 50 mg/ml TXA solution. The control group revealed an average 22% decrease in MASI, whereas, in the TXA receiving group, it decreased just a little more (29%). Moreover, hyperpigmented patches were improved on both sides in 42.5% of the TXA receiving group and 37.5% of the control group. Accordingly, addition of topical TXA with MNto the conventional triple therapy of melasma did not result in a statistically significant improvement in further reduction.^46^ Another study by Ismail et al^47^ explored the efficacy of topical vitamin C with MNin treating melasma. In this trial, thirty young women with a long history of melasma were chosen for MNof the hyperpigmented areas with a 20% solution of L-ascorbic acid. MNwas performed every 14 days six times (three months). As a result, the average MASI score dropped from 8.61 to 5.75 at the end of the three-month training, indicating that vitamin C with MNcan significantly reduce melasma hyperpigmentation.^47^ In a prospective split-face study, topical administration of PRP combined with MNexplored was compared with PRP direct injection.^12^ Although the results did not show a statistically significant difference, the MASI in the MNgroup decreased by an average of 42% compared to 30% in the injection group at the end of the follow-up. In the study conducted by Xing et al,^48^ the efficacy and safety of liposomal TXA, TXAwith MN, and conventional HQ in the treatment of melasma were compared. MASI reduction in the liposome-TXA group was 27.8%, and 33.3% in the topical TXA with MNgroup. Therefore, researchers reported that MNand liposomes are equally safe and effective for delivering TXA in treating melasma. No harm to skin barrier function or severe side effects were reported.

 An interesting study was conducted on women with bilateral melasma to investigate the superiority of topical TXA combined with MNor laser therapy.^49^ One side of the face was treated by topical TXA solution through MN approach, while the other received the same solution followed by a laser. During three months of follow-up, this procedure was performed every two weeks. At the end of the follow-up period, the reduction in MASI score was used as a primary criterion for treatment efficacy. Accordingly, participants showed a significant difference compared to the beginning of the treatment. Although, there was no remarkable difference between the results of these two methods, which indicates that both methods are suitable and effective enough.^49^

###  Iontophoresis

 Iontophoresis is a kind of electrotherapy in which a medication is administered systemically and locally by deep penetration into the tissues. It is based on the theory that positively charged ions (cations) are attracted to a negative electrode (cathode) and repel each other in an electric field. Small and hydrophilic compounds penetrate well with iontophoresis. Compared to conventional drug applications, iontophoresis may increase the possibility of transferring several hydrophilic polar molecules to the skin-deep layers up to 2000 times more.^50-52^ However, there are some restrictions toward the use of this method. Accordingly, the medications should be water-soluble, susceptible to ionization, and low dosage. In addition, iontophoresis’s therapeutic impacts may also be influenced by the tissue’s characteristics to which the electrodes are attached (e.g., thickness, permeability, and the presence of pores). It should be noted that sweat glands are the main conduit for charges to go through the skin.^53^

 Some clinical trials assessed combination of iontophoresis with some medications. Taylor et al^54^ found that vitamin C combined with a full-face iontophoresis mask can cause a significant improvement within two months in patients with melasma and post-inflammatory hyperpigmentation. The average MASI score among 35 subjects revealed a mean 15.7 improvement in abnormal pigmentations at the end of treatment. Iontophoresis of vitamin C applied to the entire face seems to be an effective short-term treatment for melasma. In addition, strict sun avoidance combined with a mandelic/malic acid skin care regimen can help maintain the treatment improvement. Another study by Arora et al^55^ compared the efficacy of 10% TXA gel applied daily combined with weekly iontophoresis to 10% TXA gel applied daily alone. In this trial, the test group received weekly iontophoresis procedures on one side of the face. Participants in the control group got TXA gel daily on both sides of their faces. According to the results, topical TXA and topical TXA combined with weekly iontophoresis therapy were well tolerated and considered safe. In addition, both sides of the face showed noticeable improvement over the three months of treatment. However, improvement appeared to be faster on the side of the face that received weekly iontophoresis (from week 3). These results confirm combination with iontophoresis as an early response for melasma treatment.^55^ The study conducted by Yoo et al^56^ evaluated the efficacy of vitamin C-iontophoresis on melasma. Ascorbic acid (vitamin C) can inhibit melanogenesis to suppress melasma. In a six-week trial, volunteers were treated twice weekly with vitamin C under a constant direct current of 0.4-0.8A by an iontophoresis device. Researchers reported a significant improvement in MASI score and no significant side effects.

 In a randomized, double-blind, placebo-controlled trial, iontophoresis of vitamin C was investigated on melasma by Hu et al^57^ Twenty-nine patients with melasma enrolled in study. On one side of face of a vitamin C solution was applied with iontophoresis, while the other side was applied distilled water as control. Twelve weeks after intervention, luminance value was decreased by 1.82 compared to control 0.58. Reported results suggest vitamin C combined with iontophoresis an effective way to treat melasma. However, 21% of patients sensed mild electrical shock and itching, 7% erythema, and 3% a burning sensation and dryness in the face, but no any serious complications were reported. Kim et al^58^ compared glycolic acid peeling and vitamin C-iontophoresis effects on melasma patients. Glycolic acid and vitamin C are depigmenting agents able to retard melanin pigment. Thirty-four individuals with facial melasma were treated with glycolic acid peeling alone or combined with vitamin C-iontophoresis (6 minutes under a constant direct current of 0.3-1.0 Ma/cm^2^). The interventions were done weekly for 12 weeks. mMASI after 12 weeks was decreased. In addition, glycolic acid pretreatment, did not affect vitamin C flux in skin. Vitamin C concentration and density in skin was related to larger flux made by iontophoresis. Another study conducted by Kyu et al^59^ investigated effects of vitamin C or multivitamin with iontophoresis on melanogenesis. Researchers first assessed vitamin C and multivitamin effects in an *in-vitro* study. Results showed these compounds reduced tyrosinase expression and microphthalmia-associated transcription factor (MITF) level, which are related to the depigmenting effects. In a clinical trial, 20 patients with melasma were enrolled for 12 weeks bi-weekly intervention. Multivitamin or vitamin C was applied on one side of face during iontophoresis. Luminance value was reduced on average by 1, suggesting both multivitamin and vitamin C iontophoresis can be effective for melasma. Some adverse effects were reported like burning sensation, erythema, and itching.

###  Laser

 Transdermal permeability can be ameliorated by using lasers. Through the photothermal effect, lasers can directly destroy the outer layers of SC, disrupt the skin barriers, and accelerate transdermal medication delivery. Skin damage from lasers heals quickly after a few days. This technique can reduce the duration of treatment and improve bioavailability. Patients with refractory melasma who did not respond to topical conventional treatments and oral anti-inflammatory peels may respond to laser and light therapy instead.^60-62^ Various lasers with different wavelengths and operational processes could be used in this context. The possibility of local irritations and the large size and inappropriateness of laser equipment for home usage restricts laser applications to specialized medical centers.^63^

 Studies evaluated effects of yttrium-aluminum-garnet (YAG) laser on melasma drug delivery. Since last five decades laser beams are being used in various processes. YAG lasers are one of most widely used types of lasers. These lasers have low mean beam power but a high beam intensity due to smaller pulse duration and better focusing behavior.^64^ Laser utility for skin disorders can cause post-inflammatory hyperpigmentation. YAG laser because of lower heat dissipation and shorter healing time can have lower risk of post inflammatory hyperpigmentation (PIH).^65,66^ Al-Dhalimi et al^67^ studied the potential therapeutic effects of using laser therapy combined with kojic acid cream to treat melasma. Their goal was to determine whether fractional erbium-doped YAG (Er:YAG) laser treatment could enhance the positive effects of topical kojic acid in participants with facial melasma. In this study, individuals were randomly assigned to receive either kojic acid cream alone on both sides or kojic acid cream alone on one side and kojic acid combined with Er:YAG fractional laser therapy on the other. MASI score and patient satisfaction index, were evaluated to assess the effectiveness of treatment. Their results showed that combining kojic acid cream and Er:YAG fractional laser can cause a statistically significant improvement over the side treated with kojic acid cream alone.^67^

 The efficacy and safety of topical HQ combined with a fractional Er:YAG laser as an additional drug delivery system for melasma treatment studied by Badawi et al.^68^ In a split-face controlled manner, thirty female participants were randomly treated with a 4% HQ cream alone on one side, daily. The other side of the face received daily 4% HQ cream followed by fractional Er:YAG laser. Eventually, mean MASI^3^ score of the combination treatment improved significantly more than HQ treatment alone. Namazi et al^69^ also concluded the same results using the Er:YAG laser combined with daily HQ 4%. However, the MASI score was not significantly affected. Therefore, one of the disadvantages of YAG laser is the need for continuous treatment (monthly), which may lead to patients’ withdrawal. Park et al^70^ combined Q-switched neodymium-doped YAG (QS-Nd: YAG) laser with glycolic acid to treat melasma. They found combination superior to laser monotherapy. Nevertheless, the laser intervention was not curative, and there was recurrence of melasma in all patients. In other hand, Wattanakrai et al^71^ conducted a study to assess the effectiveness and safety of QS-Nd:YAG laser and HQ. The researchers reported that the QS-Nd:YAG laser provided temporary improvement but had side effects such as hypopigmentation, melasma recurrence, and rebound hyperpigmentation. The research conducted by Wattanakrai et al^71^ investigated the efficiency of 1064-nm QS-Nd: YAGlaser combination with 2% HQ. Statistically significant improvement in the melasma colorimeter test and mMASI score was observed after five laser-HQ combination treatments. QS-Nd:YAG laser treatment for melasma in Asians often leads to temporary improvement but may cause side effects such as hypopigmentation, melasma recurrence, and rebound hyperpigmentation. The efficacy and safety of 585-nm Q-switched Nd:YAG laser and HQ 4% cream, was investigated by Lueangarun et al.^72^ A 585-nm Q-switched Nd:YAG laser and HQ 4% cream resulted in a more significant improvement in mMASI^4^ compared to topical treatment. However, 19% of patients experienced PIH on the laser-treated side. Laothaworn et al.^73^ At the end of the study, the combination treatment led to a significant decrease in the mMASI score, while no significant changes were observed in the treatment with laser alone. No serious adverse events were reported. In a study, Tourlaki et al^74^ investigated combination of fractional erbium-glass laser and topical TCC (HQ 4%, retinoic acid 0.03%, hydrocortisone butyrate 0.1%) in melasma. After six months, 21.1% of patients showed significant improvement, while 43.4% showed no improvement. The researchers suggest that combination therapy may benefit patients with melasma resistant to TCCalone, but its long-term effectiveness is limited. Botsali et al^75^ assessed the effectiveness of fractional Er:YAG laser-assisted TXA delivery. Er:YAG laser-assisted delivery of topical TXA 5% is an effective treatment for melasma patients resistant to conventional treatments, resulting in significantly lower mMASI scores than baseline values. In addition, Beyzayee et al^76^ investigated the effectiveness of topical methimazole 5% monotherapy versus a combination of Q-Switched Nd:YAG laser and topical methimazole 5%. No significant variance was found in reducing the mMASI score between the two groups at any point. Additionally, adverse events were also not significantly different between the two groups. In a study, Xu et al^77^ compared the effectiveness of topical 0.5% TXA solution with MNor a sham device as a control. The MNintervention was more satisfying and effective for melasma treatment, with a noticeable improvement in MASI over three months. Authors reported that MNis safe and painless without apparent side effects. Hamza et al^78^ compared the effectiveness of fractional Er:YAG laser vs. combined therapy with topical steroid. After receiving combined therapy, the MASI score significantly decreased. Additionally, the histological changes were highly significant. Lee et al^79^ studied the effects of low-fluence 1064-nm Q-switched Nd:YAG laser with or without chemical peeling using Jessner’s solution. In this study, Jessner’s peel combined with low-fluence 1064-nm Q-switched Nd:YAG laser is a safe and effective treatment for melasma in its early stages. The reduction of MASI scores was significant at eight weeks, but no significant difference was observed between the two groups at 20 weeks. Additionally, no serious adverse effects were reported using Jessner’s peeling.

 In a research performed by Bansal et al,^80^ low-fluence1064-nm Q-Switched Nd:YAG laser with topical 20% azelaic acid cream and their combination efficacy in melasma treatment were assessed. This study demonstrates that combining low-fluence laser and topical 20% azelaic acid cream is more effective in treating melasma than using either treatment alone. Erythema and burning are the reported side effects that increased by combining azelaic acid and low-influence Q-switched Nd:YAG laser. Chen et al^81^ compared the effectiveness of 1064-nm Q-switched Nd:YAG laser combined with TXA, glutathione, and vitamin C in the treatment of chloasma. Combining a 1064-nm Q-switched Nd:YAG laser with TXA, glutathione, and vitamin C can improve the therapeutic effect on patients with chloasma. It can also enhance their chloasma area, color score, and skin lesion subsidence. The treatment is safe and can regulate endocrine hormone levels. Therefore, it is suitable for clinical use. In a study, Park et al^82^ researched the tolerance and effectiveness of laser-assisted delivery of TXA, niacinamide, and kojic acid for treating melasma. Combining a low-fluence Q-switched 1064-nm Nd:YAG laser with a topical mixture of TXA, niacinamide, and kojic acid resulted in a more significant improvement in the mean hemi MASI score compared to laser treatment alone for melasma treatment. Al-Dhalimi et al^67^ studied the effectiveness of combining fractional Er:YAG laser with topical kojic acid cream for treating facial melasma. The treatment resulted in significant improvement, although mild tingling and erythema were observed on both sides of the patient. Badawi et al^68^ researched fractional Er:YAG laser-assisted drug delivery of HQ. According to our research, using fractional Er:YAG laser to deliver topical HQ through the skin is a safe and effective way to treat melasma. This technique could become essential to medical and surgical dermatology, but its high cost may limit widespread adoption. In a study conducted by Mokhtari et al^83^ fractional Er:YAG laser-assisted drug delivery of HQ in the treatment of melasma were evaluated. Treatment with HQ plus Er:YAG laser or TA injection improved hemi- mMASI and patient satisfaction. Both were equally effective, but Er:YAG had a higher recurrence rate. Wang et al^84^ studied laser-assisted delivery of TXA for melasma using a novel 1927 nm fractional thulium fiber laser. Subjects showed improved quality of life despite melasma’s psychological impact. Clinicians struggle with long-term results and recurrence prevention despite technological advancements in treatments. Saleh et al^85^ evaluated the use of Q-switched Nd:YAG laser alone or with modified Jessner chemical peeling for treating mixed melasma. They found that both low-fluence Q-switched Nd:YAG laser and modified Jessner’s peel are equally effective for treating mixed melasma. However, combining the methods is preferred for better cosmetic results and cost savings. Hawwam et al^86^ investigated the effectiveness of intradermal TXA injection alone versus intradermal TXA injection combined with Q-switched Nd:YAG laser. Combining TXA intradermal injection with low fluence Q-switched Nd:YAG laser treatment is more effective and safer for melasma, resulting in a statistically significant decrease in mMASI^4^ score compared to intradermal injection of TXA alone after 12 weeks of treatment and at the 24-week follow-up. The combination treatment also results in fewer side effects. Vasanop et al^87^ compared the effectiveness of low-influence Q-switched neodymium-doped YAG1064 with or without glycolic acid peeling. Throughout the study period, there was a decrease in the mean relative lightness index (RL*I) on the combined treatment side. At the fourth-week follow-up, the maximum improvement was observed, reduction. Results demonstrated that combining low-fluence Q-switched Nd-YAG laser with glycolic acid peeling can increase adverse events like burning, erythema, and hypopigmentation. Colorimeter adverse events got better except for hypopigmentation, which remained unchanged.

**Figure 6:**
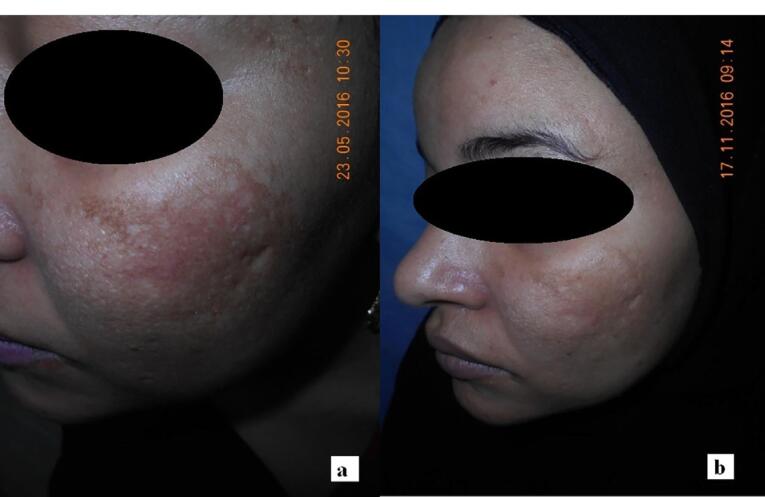
Improvement in melasma with fractional Carbon dioxide laser and intradermal Tranexamic acid pretreatment (a) and post-treatment. Reprinted with permission from Tawfic et al^94^

 CO_2_ laser is a widely used laser in dermatology due to its mid-infrared wavelength of 10600 nm that is well absorbed in water. This makes it ideal for precise, safe ablation with good hemostasis on skin, which contains a very high-water percentage. CO_2_ laser is effective in ablating benign raised lesions and is used in esthetic dermatology for revising acne scars and photorejuvenation. Recent advancements in CO_2_ laser technology have resulted in smaller spot sizes and improved precision for laser surgery. This has led to greater flexibility in tip sizes and treatment protocols for fractional CO_2_ laser procedures. Dermatological applications of the CO_2_ laser are projected to expand further in the future.^88,89^ Elmorsy et al^90^ compared low-power fractional CO_2_ laser followed by Jessner’s peel versus Jessner’s peel alone. The group that used a combination of fractional laser and Jessner’s solution experienced faster improvement and higher patient satisfaction. Both groups had melasma recurrence at follow-up, indicating the condition requires ongoing management. Qu et al^91^ demonstrated the significant difference between the MASI score of patients treated with TXA twice daily compared to treatment with administration of TXA twice daily followed by fractional CO_2_ laser every week. In this trial, 90 people with bilateral melasma were selected and divided into two equal groups. After a three months follow-up, a substantial difference between the laser-receiving group and the control group was observed. Authors also reported that low-power fractional CO2 laser is safe and effective, and TXA can reduce dilation of blood vessels and avoid post-inflammatory hyperpigmentation. In a study, Trelles et al^92^ compared the effectiveness of creams alone, CO_2_ fractional ablative resurfacing alone, or a combination of the two for treating melasma. MASI scores significantly improved with fractional CO_2_ laser and topical Kligman formula compared to monotherapy at 6 and 12 months, resulting in well-maintained results for melasma treatment. Nourmohammadi Abadchi et al^93^ investigated the effectiveness of topical HQ with fractional CO_2_ laser or HQ monotherapy as a control. The intervention reduced the darkness and homogeneity of hyperpigmentation combination therapy. Although laser and HQ can be applied to obtain earlier positive results, it has high cost and no difference in efficiency with the control group, and it is not recommended for long-term treatment. Tawfic et al^94^ compared the effectiveness of low-power fractional CO_2_ laser alone or combined with TXA. Both groups showed improvement in MASI, superior for integrated CO_2_ laser and TXA (Figure 6). The authors reported that combination therapy is safe, and no severe side effects were reported, except for pain or slight discomfort after the sessions. In another study, Tawfic et al^95^ investigated the efficiency of low-power low-density fractional CO_2_ laser with TXA microinjection or TXA microinjection alone. No significant difference was observed in MIlevel and percentage of improvement. To treat melasma, injecting TXA alone or combined with a low-power, low-density fractional CO_2_ laser in a sequential pattern is both practical and safe. It is recommended to use the combined treatment. Dermoscopy is an important non-invasive tool to evaluate melasma and track patients’ response to treatment.

 Newer lasers such as diode and long-pulsed Nd:YAG are safe for dark-skinned patients in dermatology. Diode lasers are highly efficient, small, and portable, with wavelengths of 800-815 nm and 10-50 millisecond pulse.^96^ Monique et al^13^ investigated the effectiveness and safety of topical 1927-nm diode laser with topical HQ 2% cream or moisturizer. The MoPASIscore showed 50% improvement after four weeks for the HQ group and 12 weeks for the moisturizer group. Authors reported that the non-ablative, fractional, 1927-nm diode laser produced significant improvement in hyperpigmentation which was faster than other groups. A picosecond laser emits extremely short pulses of light in the picosecond range, making them unique in their ability to deliver high peak power and generate intense bursts of energy. Their short pulse duration makes them well-suited for various field applications, including tattoo removal, skin rejuvenation, and treating pigmented lesions in medicine. Pro yellow laser, or the picosecond yellow laser, is used in dermatology for various skin conditions. It emits laser light at a specific wavelength of 532 nm, which appears yellow.^97,98^ Choi et al^99^ compared 7 week 2% HQ (daily) and 5 week 750 picosecond laser using 1064 and 595 nm (weekly) versus 7 week 2% HQ alone. Picosecond laser and 2% HQ had a better effect than 2% HQ alone (76.92%). No serious adverse events occurred. The dual-wavelength picosecond laser used in this study reduces the photothermal impact during pigment removal and the procedure duration, thus reducing adverse events. In another research by Manuskiatti et al,^100^ combination of a 755-nm picosecond laser and HQ 2% cream versus HQ 2% cream alone for the treatment of melasma were evaluated. Both sides showed significant improvement in MASI scores. However, 15% of patients on the laser-treated side experienced mild post-inflammatory hyperpigmentation, while no adverse effects were reported on the HQ monotherapy side. Additionally Li et al^101^ investigated 755-nm picosecond alexandrite laser with topical TXA versus laser monotherapy for melasma and facial rejuvenation. Significant improvements in hemi-MASI, dyschromia, and skin texture on both halves were observed through the follow-up. Combination therapy showed better results than monotherapy in hemi-MASI and dyschromia at 1- and 3-month follow-ups. Laser monotherapy reduced redness and sensitivity during the 7-day post treatment recovery period. The picosecond alexandrite laser and topical TXA combination therapy showed synergistic efficacy for hemi-MASI and dyschromia improvements over laser monotherapy. Chalermachi et al^102^ compared the fractional picosecond 1064 nm laser plus 4% HQ cream with the use of HQ cream only. The study found that the picosecond laser caused temporary mild skin redness, peeling, and burning, but all side effects resolved independently without treatment. In addition, Mohamed et al^103^ compared efficacy of 577 nm pro-yellow laser with or without 4% HQ in the treatment of melasma. After six months of treatment, the MASI score was significantly lower on the laser-treated side than the non-laser-treated side, indicating that adding a 577 nm pro-yellow laser to melasma treatment can help maintain improvement and reduce recurrence rates.

 Erbium glass fiber lasers are utilized in dermatology to treat various skin conditions, such as skin resurfacing, pigmentation disorders, and scar revision. These lasers use a specific glass doped with erbium ions, emitting laser light when stimulated with an appropriate energy source. One of the advantages of erbium glass fiber lasers is their ability to accurately target specific areas of the skin, minimizing damage to surrounding tissues.^104,105^ In a study, Tourlaki et al^74^ investigated combination of fractional erbium-glass laser and topical TCC(HQ 4%, retinoic acid 0.03%, hydrocortisone butyrate 0.1%) in melasma. After six months, 21.1% of patients showed significant improvement, while 43.4% showed no improvement. The researchers suggest that combination therapy may benefit patients with melasma resistant to TCC alone, but its long-term effectiveness is limited. Thulium fiber laser is an infrared laser used for various dermatological treatments, such as skin rejuvenation, fractional resurfacing, pigmented lesion removal, and vascular lesion treatment. It offers precise targeting, controls treatment, and minimizes damage to the surrounding skin.^106^ Wantiphakdeedecha et al^107^ researched the use of thulium 1927-nm fractional laser-assisted topical TXA delivery in treating facial melasma. By the 6th month, notable variances in MI and mMASI scores from the initial measurements were still observed, except in the MI for controls. No severe adverse events were documented for either group. Administering TXA through fractional thulium laser (FTL) over four weeks is a safe and efficient melasma treatment, resulting in substantial improvement for up to 3 months. The findings also propose that a repeated regimen every three months could be beneficial for treating persistent melasma.

###  Radiofrequency (RF)

 Microwave technology has attracted much attention in transdermal applications in recent years. Microwave technology enhances transdermal medication delivery by temporarily loosening the skin barriers and permitting the drugs to penetrate the lipid structure of the SC.^108^ In an uncontrolled prospective study, Cameli et al^109^ found a substantial reduction in MASI score and erythema in patients treated with six weekly sessions of monopolar RF and 1% kojic acid. After six months of follow-up, only 4% of patients experienced relapse. It was much milder than the primary state, and no adverse side effects were reported.

 According to Kwon et al^108^ study, low fluence Q-switched Nd:YAG laser paired with RF and MNwas more effective than QS Nd:YAG laser alone in treating melasma. Although there was no significant difference in treatment-related adverse reactions between the two groups in the trial, the incidence of mottle hypopigmentation and reversible hyperpigmented patches was higher in the group receiving QS Nd:YAG treatment alone.^108^ Gulfan et al^110^ compared the noninsulated micro-needle paired RF with or without polynucleotides. On the basis of their study, combining of micro-needle paired RF with polynucleotide is not superior to micro-needle paired RF without polynucleotide in treatment of melasma. Moreover, they found micro-needle paired RF as a safe and effective method to improve skin roughness that could be used as an adjunctive treatment for melasma. In a study, Norma et al^109^ compared the effectiveness of topical monopolar RF with 1% kojic acid. This study presents the first report of an improvement in melasma through the combined use of monopolar RF with transdermal delivery of depigmenting agents. This method could be a safe, well-tolerated, and effective alternative for treating melasma.

###  Sonophoresis

 Sonophoresis (phonophoresis) is known as applying acoustic waves on the skin surface to facilitate transdermal delivery through cavitation (the formation and oscillation of microbubbles), acoustic streaming, thermal effects, and direct ultrasound pressure that affects the SC keratinized structure.^111^ Non-invasiveness, minimal skin damages, ease of use, and a high degree of universality are among the essential advantages of sonophoresis.^18,111,112^

 The efficacy of 694-nm fractional Q-switched ruby (QSR) laser and vitamin C sonophoresis in the treatment of melasma was explored in a study by Zhou et al.^113^ The study enrolled 26 patients with melasma diagnosis who were first treated with laser therapy and then 20% vitamin C solution applied with sonophoresis to their faces every two weeks. The MASI score compared to the beginning of therapy and the side effects were examined after each session. Accordingly, the MASI score reduced at the end of the treatment, and no adverse effect was observed in the patients.^113^

 In a randomized split-face study for the treatment of melasma by Vachiramon et al,^114^ the efficacy and tolerability of using single-blinded ultrasonography were investigated. Individuals applied 2% HQ daily to one side of their face, while the other side received 2% HQ daily, followed by ultrasound treatment every month. Results indicated minimal adverse effects with this method. However, there were no discernible differences between the two sides at the end of the follow-up.^114^

 Pietro et al^109^ studied emulsion containing ascorbic, azelaic, and kojic combined with low frequency sonophoresis in patients with melasma and solar lentigo. The degree of depigmentation among 48 subjects revealed a good improvement at the end of treatment. Researchers have found that using ultrasound with a gentle technique can improve the effectiveness of topical agents in treating cosmetic skin disorders. Combining drugs with a suitable vehicle and low-frequency sonophoresis can also aid in penetration of high molecular weight compounds. Some clinical trials compared between physical methods, which we explained before.

 In a study conducted by Mekaway et al,^49^ efficacy and safety of TXA combined with fractional CO_2 _laser or MNin the treatment of melasma were evaluated. No significant difference in MASI reduction between two methods was reported. Therefore, researchers reported that MNand fractional CO_2_ lasers are equally safe and effective for delivering TXA to treat facial melasma. Adverse effects in both methods were erythema, burning, swelling, blistering, crust, and post-inflammatory hyper or hypo-pigmentation. Another study by Ghandehari et al^115^ compared the efficacy and safety of TXA combined with fractional CO_2 _to TXA with MN. According to the results, there was no significant difference between the two methods till day 150, and both sides of the face showed noticeable improvement over the five months of treatment. Adverse effects were temporary, with no difference between both sides except for erythema that last longer with MN.

 Debasmita et al,^116^ studied topical 3% TXA with MNor Q-switched Nd:YAG laser. In this study, MNand QS Nd:YAG laser combined with topical 3% TXA gel were equally effective in treating melasma. Both interventions had side effects, including erythema, pain, and burning sensation. However, MNhas more downtime and may be less preferable as a lunchtime procedure. Patient satisfaction scores were not significantly different between the two groups. Another study conducted by Balevi et al,^117^ the efficacy and safety of QS-Nd:YAG laser combined with MNfor topical administration of vitamin C were assessed. QS-Nd:YAG laser treatment enhances dermal blood circulation and promotes vitamin C absorption. This combination therapy shows promise for treating stubborn melasma. Sobhi et al,^118^ investigate effects of glycolic acid 70% peels or liposomal vitamin c iontophoresis on melasma. Both sides were improved, but the side treated with liposome vitamin C showed better results. Side effects were few and transient. We concluded that the liposomal form of vitamin C is a new, safe, effective, easy, and painless method of treating melasma. Lee et al,^119^ researched the efficacy and safety of 1064-nm Q-switched Nd:YAG laser toning and an enhanced effect of ultrasonic application of vitamin C on melasma. Using a combination of 1064-nm QS-Nd:YAG laser and ultrasonic application of vitamin C can effectively treat complex facial pigmentation problems like melasma without any post-inflammatory hyperpigmentation or rebound during a 3-month follow-up period. Alexiades et al^120^ conducted a randomized, double-blind, split-face study to evaluate the efficacy and safety of Fractional Ablative Er:YAG laser mediated transdermal delivery of cosmetic agents and a novel acoustic pressure ultrasonic treatment of melasma, skin aging, and acne scars. In a clinical study, combining ultrasound treatment with fractional ablative Er:YAG laser facilitated trans epidermal delivery of topical anti-aging actives proved safe and effective in improving skin conditions such as rhytids, melasma, and acne scars. Statistically, the ultrasound side showed a more significant improvement in pigment levels than the other treatment methods. Also, there was a study combining physical method with nanocarriers. Table 1 provides an overview of the various physical methods and their effects on melasma.

**Table 1:** Details of physical energy-based methods clinical trials used in melasma tretment.

**Method type**	**Applied medication**	**Sample size**	**Treatment duration **	**Treatment follow-up **	**Clinical outcome**	**Ref.**
Micro-needle patch	4-n-butylresorcinol (Test), placebo (Control)	45	3 days interval 4 days interval	2 months	The MI in the 3-day interval test decreased more than in the 4-day interval test. In the 4-day interval test: the MI for the test group decreased to 1.56% and 3.57% at 4 and 8 weeks. The MI for the control group decreased to 0.94% and 1.78%.	^22^
	Hyaluronic acid with niacinamide, ascorbic acid 2-glucoside, TXA, resveratrol, 4-n-butyl-resorcinol, and Halidrys siliquosa extract	20	3 months	1 month	The L values decreased from 59.18 ± 3.32 to 60.29 ± 2.25	^30^
	Anti‐acne ingredients DMN patches	30	28 days	3,7 and 28 days	The patch reduced acne volume by 12.34% in 3 days and an additional 10.01% after 7 days of continuous use. After 28 days of treatment, there was a 5.88% decrease in skin melanin.	^23^
Micro-needling	TXA	20	3 months	1 month	The MASI score in the group with TXA was an average reduction of 1.4	^46^
	Vitamin C	30	6 sessions every 2 weeks	3 months	MASI score decreased from 8.61 ± 4.45 to 5.75 ± 4.16	^47^
	Topical PRP + MN (A), Topical PRP + Mesoneedles (B)	23	3 sessions every month	1 month	(A): MASI score decreased from 6.13 ± 2.73 to 3.57 ± 2.42 (B): MASI score decreased from 5.73 ± 2.77 to 3.40 ± 2.49	^12^
	TXA + MN (A), TXA + Mesoneedles(B)	30	0, 4 and 8 weeks	5 months	(A): MASI score decreased from 9.11 ± 4.09 to 5.06 ± 2.14 (B): MASI score decreased from 6.93 ± 2.16 to 4.45 ± 1.69	^36^
	TXA + MN (A), MN (B)	40	2, 4, and 6 weeks	2 months	(A): MASI score decreased from 4.31 ± 1.77 to 1.471 ± 1.12 (B): MASI score decreased from 3.89 ± 1.96 to 3.090 ± 1.664	^37^
	MN + 5% retinoic acid (A), 5% retinoic acid (B)	42	15, 30, 45, and 60 days	2 months	(A): MASI score decreased from 4.31 ± 1.77 to 1.471 ± 1.12 (B): MASI score decreased from 3.89 ± 1.96 to 3.090 ± 1.664	^39^
	Trichloroacetic acid + MN (A), Trichloroacetic acid (B)	40	(A): 4 sessions monthly (B): 8 sessions bimonthly	3 months	The MASI score in Group A was significantly higher than Group B	^45^
	TXA + MN (A), intradermal TXA injection (B)	56	6 sessions at 2 weeks intervals.	3 months	(A): MASI score decreased from 13.83 ± 7.23 to 3.65 ± 2.32 (B): MASI score decreased from m 13.83 ± 7.23 to 3.49 ± 2.91	^38^
	YAG laser + vitamin C + MN (A), YAG laser (B)	16	4 sessions at 4 weeks intervals	6 months	(A): MASI score decreased from 7.04 ± 4.55 to 2.49 ± 2.30 (B): MASI score decreased from 6.13 ± 4.94 to 4.52 ± 3.49	^117^
	TXA + MN, Vitamin C	30	2, 4, and 6 weeks	6 weeks	The MASI score showed significant improvement in both TXA and vitamin C	^121^
	Topical 3% TXA gel + YAG laser (A), topical 3% TXA gel + MN (B)	60	(A): YAG laser sessions monthly with daily 3% TXA gel	Topical 3% TXA gel + YAG laser (A), topical 3% TXA gel + MN (B)	(A): mMASI score decreased from 5.12 ± 2.66 to 2.33 ± 1.33 (B): mMASI score decreased from 4.60 ± 2.38 to 1.88 ± 1.08	^116^
	Topical 4% HQ (A), MN + topical 4% TXA (B)	50	8 weeks	8 weeks	(A): MASI score decreased from 6.604 ± 4.02 to 3.032 ± 1.19 (B): MASI score decreased from 6.348 ± 3.84 to 3.712 ± 1.19	^44^
	Methimazole 5% cream (A), placebo cream (B)	30	12 sessions every week	3 months	(A): MASI score decreased from 7.98 ± 2.53 to 5.03 ± 2.77 (B): MASI score decreased from 6.82 ± 3.13 to 6.87 ± 3.21	^43^
Micro-needling	TXA + MN (A), MN (B)	42	6 sessions at 2-week interval	2 weeks	(A): MASI score decreased from 6.4-25.2 to 1-18 (B): MASI score decreased from 6.4-22.5 to 6-21	^42^
	Topical TXR, vitamin C	20	6 sessions 2 weeks	3 months	The MASI score decreased following treatment.	^41^
	Micro-needle paired RF + polynucleotides (A), micro-needle paired RF (B)	30	every 2 weeks	6 months	The mMASI scores for groups (A) and (B) showed no statistically significant differences.	^110^
	Topical 0.5% TXA + MN (A), TXA (B)	30	once per week for 12 weeks.	3 months	(A): MASI score decreased from 43.33 ± 5.52 to 36.48 ± 4.84 (B): MASI score decreased from 41.97 ± 5.83 to 40.56 ± 5.27	^122^
	Topical 4% TXA + MN (A), 4% HQ	60	4,8 and 12 weeks	3 months	(A): MASI score decreased from12.89 ± 5.16to 6.84 ± 4.31 (B): MASI score decreased from 13.56 ± 4.88 to 7.16 ± 4.38	^40^
	Triple combination cream(A), triple combination cream + MN (B), triple combination cream + TXA (C), triple combination cream + MN + TXA (D)	64	2 sessions at 1-month interval	2 months	The groups showed a 24% reduction in epidermal melanin density. There was a 25% reduction in pendulum melanocytes, especially in the MN and TXA group, which presented a 41% reduction.	^123^
	MN + Vitamin C, PRP	10	6 sessions	1 month	MN with vitamin C was more effective than PRP	^124^
	MN + Vitamin C (A), MN + TXA (B)	30	every 2 weeks	4 months	(A): MASI score decreased from 6.34 ± 3.78 to 3.04 ± 2.64 (B): MASI score decreased from 5.98 ± 3.58 to 3.64 ± 2.62	^125^
	1.8% liposomal TXA (A), MN + TXA solution (B), topical 2% HQ cream (C)	60	(A): twice a day (B): every week (C): every night	3 months	(A): MI score decreased from 203.14 ± 10.27 to 78.33 ± 11.41 (B): MI score decreased from 261.13 ± 17.89 to 221.17 ± 18.83 (C): MI score decreased from 214.85 ± 10.19 to176.48 ± 9.57	^48^
	TXA + MN (A), TXA + YAG laser (B)	30	6 sessions twice-weekly sessions	2 weeks	(A): MASI score decreased from 3.43 ± 1.84 to 1.59 ± 1.51 (B): MASI score decreased from 3.51 ± 1.84 to 1.78 ± 1.51	^49^
	TXA + MN (A), TXA + YAG laser (B)	30	3 months	1-3 months	Group A had a better MASI score on day 60, but no differences between the two methods were observed on days 30, 90, and 150.	^115^
Iontophoresis	Vitamin C	15	6 weeks	6 weeks	The MASI score showed a significant clinical improvement in treating melasma.	^56^
	Vitamin C	35	2 months	54 months	15.7% MASI score improvement compared to the baseline	^54^
	nanosome vitamin C iontophoresis (A), glycolic acid peel 70% (B)	14	6 sessions	6 sessions	(A): MASI score decreased from 8.31 ± 2.81 to 4.77 ± 2.79 (B): MASI score decreased from 7.97 ± 2.53 to 6.21 ± 2.72	^118^
	Vitamin C	29	4 weeks	3 months	the MASI score may be incorrect in assessing the exact effects on melasma but L value decrease from 4.60 to 2.78.	^118^
	Vitamin C-iontophoresis, 30% glycolic acid	34	12 weeks	3 months	The mean scores of both mMASI were lower than the baseline in all groups.	^58^
	Multivitamin, vitamin C	20	12 weeks	3 months	No significant difference between multivitamins and vitamin C and L value was decreased in both.	^58^
	TXA GEL10% + iontophoresis, TXA GEL10%	30	12 weeks	3 months	Iontophoresis treatment reduced the melasma area by 5 weeks, while topical TXA took 7 weeks to show results.	^55^
Sonophoresis	Vitamin C sonophoresis + QSR laser	26	6 session at 2-week intervals.	3 months	The mean MASI score decreased from 15.51 ± 3.0 to 10.02 ± 4.39	^113^
	2% HQ (A), high-intensity focused ultrasounds (B)	25	3 sessions at monthly intervals	20 weeks	(A): The MASI score decreased from 15.00 ± 6.19 to 13.52 ± 6.22 (B): The MASI score decreased from 15.33 ± 5.91 to12.52 ± 6.91	^114^
	YAG laser + ultrasonic topical vitamin C	8	4 sessions at 1 interval.	3 months	The mean MASI score of the combination treatment improved significantly more than laser treatment alone.	^119^
	Fractional ablative Er: YAG laser + topical agents	15	2 monthly	1, 3, and 6 month	Ultrasound treatment significantly improved pigment levels more than FELR and topical agents alone.	^120^
	Treated by emulsion + LFS (low frequency sonophoresis) (A), emulsion (B), placebo + LFS (C)	48	2-times a week	5 weeks	The more remarkable results were obtained for melasma treated with emulsion and LFS (group A). Depigmentation occurred to a lesser extent in group(B) and group(C).	^126^
Laser	YAG laser + kojic acid, YAG laser	25	6 sessions at 2 week intervals	3 months	MASI score of the combination of YAG laser and kojic acid cream showed a statistically significant improvement than the side treated with kojic acid alone.	^67^
	YAG laser + 4% HQ cream (A), 4% HQ cream (B)	30	6 sessions at 2 week intervals	3 months	(A): The MASI score decreased from 9.31 ± 2.84 to 3.74 ± 2.86 (B): The MASI score decreased from 9.1 ± 2.70 to 6.82 ± 2.97	^68^
	YAG laser + 4% HQ cream (A), 4% HQ cream (B)	29	3 sessions at 4 week intervals	7 months	(A): The MASI score decreased from 17.64 ± 8.21 to 8.35 ± 4.87 (B): The MASI score decreased from 18.43 ± 8.62 to 10.82 ± 5.95	^69^
	Fractional CO_2_ laser + topical TXA	90	4 sessions at 3-week intervals	3 months	(A): The MASI score decreased from 21.2 ± 1.7 to 15.4 ± 1.5 (B): The MASI score decreased from 20.7 ± 1.8 to 17.2 ± 1.9	^91^
	Fractional CO_2_ laser + TXA (A), MN + TXA (B)	30	6 sessions at 2 week intervals	3 months	(A): The MASI score decreased from 3.51 ± 1.84 to 1.78 ± 1.51 (B): The MASI score decreased from 3.43 ± 1.84 to 1.59 ± 1.51	^49^
	Topical HQ 4% + fractional CO_2_ laser (A), topical HQ 4% (B)	40	3 sessions at 3 week intervals	3 months	(A): The hyperpigmentation of Darkness decreased from 3.8 ± 0.82 to 2 ± 1.70 (B): The hyperpigmentation of Darkness decreased from 3.6 ± 0.95 to 1.98 ± 1.00	^93^
	Fractional CO_2_ laser + Topical TXA (A), CO_2_ laser + intradermal TXA (B), CO_2_ laser (C)	30	5 sessions every 4–6 weeks		(A): The MASI score decreased about 1.70 ± 2.62 (B): The MASI score decreased about 1.45 ± 1.89 (C): The MASI score decreased about 1.75 ± 2.15	^94^
	Fractional CO_2_ laser + TXA, TXA alone	29	3 sessions every 6 weeks	4 months	Study intervention Showed a significant reduction post-treatment on both sides of the face. But No statistically significant difference was found between the two groups.	^95^
	Nonablative fractional diode laser + HQ 2% cream (A), moisturizer (B)	40	2-week intervals	4 and 12 weeks	(A): MoPASI score decreased from 5.7 ± 1.2 to 3.5 ± 1.2 (B): MoPASI score decreased from 5.7 ± 1.6 to 3.8 ± 1.0	^13^
	Low-fluence QS-Nd: YAG Laser + Glycolic Acid 30% (A), Low-fluence QS-Nd: YAG Laser (B)	15	5 sessions	3 months	(A): The MASI score decreased from 21.2 ± 1.7 to 15.4 ± 1.5 (B): The MASI score decreased from 20.7 ± 1.8 to 17.2 ± 1.9	^87^
	Oral TXA (A), TXA + topical HQ 4% (B), TXA + YAG laser (C)	60	every 3 weeks for 3–2 sessions	9 months.	(A):The MASI score decreased from 9.63 ± 3.64 to 8.93 ± 3.83 (B):The MASI score decreased from 8.80 ± 3.68 to 6.70 ± 3.36 (C):The MASI score increased from 8.95 ± 4.64 to 8.99 ± 4.69 (B) had the most improvement.	^127^
	YAG laser + topical 2% HQ (A), YAG laser (B)	22	5 sessions at 1 week intervals	3 months	(A): The MASI score decreased from 22.3 ± 1.8 to 5.7 ± 0.8 (B): The MASI score decreased from 21.9 ± 1.8 to 16.6 ± 1.4	^71^
Laser	QS-Nd: YAG laser (A), QS-Nd: YAG laser + Oral TXA (B)	48	(A),(B)2 sessions (B) 8 weeks	1 month	(A): The MASI score decreased from 7.9 ± 3.9 to 6.0 ± 3.2 (B): The MASI score decreased from 8.0 ± 4.3 to 5.1 ± 3.3	^128^
	QS-Nd: YAG laser + 4% HQ cream, topical 4% HQ cream	21	5 sessions at 2-week intervals	5 months	The mMASI score showed a more significant improvement in the combination-treated than topical-treated after 2, 4, and 8 weeks.	^72^
	YAG laser + 30% glycolic acid (A), YAG laser (B)	16	GA: 3 sessions at 2-week intervals laser: 6 sessions at 1-week intervals	5 months	(A): The MASI score decreased from 21.2 ± 1.7 to 15.4 ± 1.5 (B): The MASI score decreased from 20.7 ± 1.8 to 17.2 ± 1.9	^70^
	Topical 3% TXA + YAG laser (A), YAG laser (B)	25	TXA: 8 weeks laser: 4 weeks	2 months	(A): The MASI score decreased from 16.38 to15.2 (B): The MASI score decreased from 16.48 to 15.88	^73^
	Picosecond laser (ps) + HQ 2%, HQ 2% cream	20	HQ: 4 weeks laser: Every two weeks.	1,3,and 6-months	MASI scores significantly improved with no significant difference between HQ alone and HQ + laser.	^100^
	Erbium-glass laser + TCC	76	For 10 days, use it daily and then have four laser treatments done at 3-week intervals.	6 months	The MASI score decreased from 1.75 ± 7.926 to 6.349 ± 5.842 after 1 month, then increased to 13.04 ± 7.822 after 6 months from the last laser session.	^74^
	Oral TXA + topical TXA + YAG laser (A), topical TXA + YAG laser (B)	34	laser:4biweekly sessions	3 months	(A): The mean MASI score decreased from 4.4 ± 4.2 to 1.6 ± 1.5 (B): The mean MASI score decreased from 2.7 ± 2.0 to 1.6 ± 1.7	^75^
	Topical methimazole 5% (A), YAG Laser + topical methimazole 5% (B)	27	6 sessions	3 months	(A): The mean MASI score decreased from 5.07 to 3.24 (B): The mean MASI score decreased from 5.02 to 2.71	^76^
	Fractional CO_2_ Laser + TXA (A), YAG Laser + TXA (B)	30	3 months	3 months	(A): The mean MASI score decreased from 4.89 to1.84 (B): The mean MASI score decreased from 5.14 to 2.52 Group (A)showed significant improvement compared to group(B)	^129^
	Oral TXA (A), YAG laser + oral TXA (B)	60	every 2 weeks	3 months	The mean MASI score of the combination treatment improved significantly more than oral TXA treatment alone.	^77^
	YAG laser (A), YAG laser + topical steroid (B)	22	6 sessions	3 months	(A): The mean MASI score decreased from 21.5 ± 9.8 to 11 ± 5.3 (B): The mean MASI score decreased from 21.5 ± 9.8 to 6.8 ± 2.1	^78^
	YAG laser (A), YAG laser + Jessner’s solution(lactic acid, salicylic acid, resorcinol and ethanol) (B)	52	10 sessions in two-week intervals	5 months	(A): the mean MASI score decreased from 8.68 ± 4.06 to 8.60 ± 3.88 (B): the mean MASI score decreased from 8.98 ± 3.72 to 7.13 ± 2.57	^79^
	Low-fluence QS-Nd: YAG laser (A), 20% azelaic acid cream(B),low-fluence QS-Nd: YAG laser + 20% azelaic acid cream (C)	60	6 and 12 weeks	3 months	(A): the mean MASI score decreased from 21.11 ± 6.91 to 10.11 ± 4.28 (B): the mean MASI score decreased from 15.90 ± 5.49 to 9.68 ± 3.37 (C): the mean MASI score decreased from 18.73 ± 7.53 to 4.94 ± 1.67	^80^
	Picosecond laser + 4% HQ (A), 4% HQ (B)	30	4, 8 and 12 weeks	3 months	(A): the mean MASI score decreased about 4.18 ± 2.03 B): The mean MASI score decreased from about 3.52 ± 1.4	^102^
	Picosecond alexandrite laser + topical TXA,laser	37	4–5 weeks intervals	6 months	Group(A) demonstrated synergistic efficacy for MASI and dyschromia improvements over laser monotherapy.	^101^
Laser	Picosecond laser + 2% HQ (A), 2% HQ (B)	39	HQ: 7 weeks laser: 5 weeks	18 weeks	(A): the MASI score decreased from 22.29 to 5.68 (B): the MASI score decreased from 21.94 to 16.61	^99^
	Factional laser + topical TXA, laser + normal salin	46	4 weeks	6 months	MASI scores showed no significant difference between TXA and control sides at 3 months, but a significant difference was observed at 6 months.	^107^
	HQ 4% + pro-yellow laser (A), HQ 4%(B)	82	3 sessions	6 months	(A): the MASI score decreased from 7.26 ± 4.76 to 1.16 ± 1.8 (B): the MASI score decreased from 7.31 ± 4.76 to 2.54 ± 2.45	^103^
	YAG laser (A), YAG laser + TXA + glutathione + vitamin C (B)	60	every two weeks.	6 weeks	(A): the MASI score decreased from 11.28 ± 2.19 to 6.23 ± 2.05 (B): the MASI score decreased from 11.47 ± 2.21 to 2.74 ± 1.36	^81^
	YAG laser + TXA + niacinamide + and kojic acid, YAG laser	25	5 sessions at 2-week intervals	3 months	The mean MASI score of the combination treatment improved significantly more than laser treatment alone.	^67^
	YAG laser + kojic acid, kojic acid	25	6 sessions at 2 week interval.	3 months	The mean MASI score of the combination treatment improved significantly more than kojic acid treatment alone.	^67^
	YAG laser + HQ 4% (A), HQ 4% (B)	30	6 sessions at 2-week intervals.	3 months	(A): the MASI score decreased from 9.31 ± 2.84 to 3.74 ± 2.86 (B): the MASI score decreased from 9.1 ± 2.70 to 6.82 ± 2.97	^68^
	TXA + topical HQ (A), YAG laser + topical HQ (B)	31	3 months	3 months	The MASI score for group A showed significant improvement compared to group B.	^83^
	Thulium fiber laser + topical TXA	10	7 days	6 months	The mean MASI score decreased about 2.5	^84^
	Kligman’s topical cream (A), CO_2_ fractional laser (B), Both A, B (C)	30	1,2,6,12 months	12 months	The MASI scores of Group C showed significant improvement compared to the scores of Group A and B.	^92^
	Gessner’s solution + YAG laser, YAG laser	19	6 sessions	1 month	The MASI score was significantly decreased, with no significant difference between them.	^85^
	YAG Laser + HQ 4% (A), HQ 4% (B)	29	3 sessions at 4-week intervals	7 months	(A): the MASI score decreased from 17.64 ± 8.21 to 8.35 ± 4.87 (B): the MASI score decreased from 18.43 ± 8.62 to 10.82 ± 5.95	^69^
	TXA (A), YAG laser + intradermal injection TXA (B)	40	3 months	6 months	The mean MASI score of the combination treatment improved significantly more than the TXA treatment alone.	^86^
	Fractional CO_2_ laser + Jessner’s solution (A), Jessner’s solution (B)	40	6 sessions	3 months	(A): the mean MASI score decreased from 6.01 ± 4.51 to 4.22 ± 4.26 (B): the mean MASI score decreased from 4.42 ± 3.89 to 2.69 ± 2.46	^90^
Radiofrequency	Monopolar RF + topical 1% kojic acid phytocomplex	50	6 sessions at 1-week intervals	1and 6 months	The MASI score showed that hyperpigmentation was significantly reduced.	^109^

###  Nanocarriers and nanoparticles 

**Figure 7:**
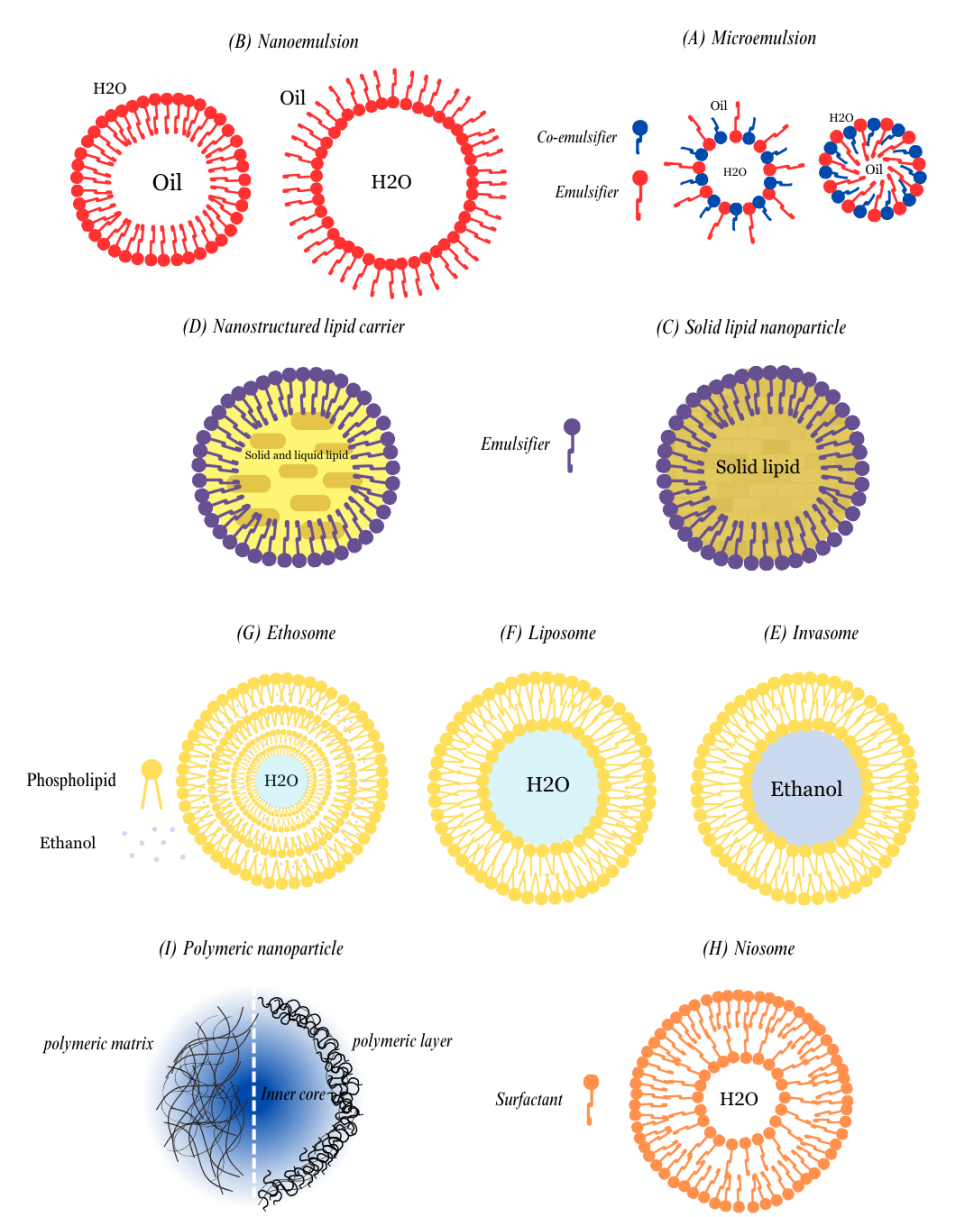
Nanocarriers and nanoparticles including: (A) microemulsion, (B) nanoemulsion, (C) solid lipid nano particle, (D) nanostructured lipid carriers, (E) invasome, (F) liposome, (G) ethosome, (H) niosome, (I) polymeric nanoparticle

 Topical hypopigmented drugs have been successfully delivered to the deep layer of skin using various methods, including encapsulation in nanocarrier-based delivery systems. Colloidal systems with an average diameter of 500 nm or less are called nanocarriers. The most recent studies on novel nanocarriers for cutaneous and transdermal drug administration focused on MEs, NEs, LNPs, SLNs, NLCs, liposomes, niosomes, ethosomes, and invasomes (Figure 7). Using nanotechnology in drug delivery for the treatment of hyperpigmentation provides several advantages. Nano drug delivery systems offer targeted and effective solutions for managing this skin condition. The benefits include:

A) Enhanced skin penetration: Nanoparticles can deliver active ingredients deeper into the skin and reaching the melanin-producing cells more effectively. This results in improved treatment outcomes for hyperpigmentation.
B) Reduced irritation: Nano drug delivery can minimize skin irritation and side effects by encapsulating active compounds, allowing for controlled and gradual release.
C) Precise targeting: Nanocarriers can specifically target melanin-producing cells, leading to a more focused treatment that reduces the risk of uneven skin tone or further pigmentation.
D) Improved stability: Nanoparticles enhance the stability of active ingredients, preventing degradation and maintaining the efficacy of the treatment over time.
E) Customized formulations: Nanotechnology allows for tailored formulations, enabling healthcare professionals to create personalized treatments based on individual patient needs.

 These advantages underscore the potential of nanotechnology in addressing hyperpigmentation concerns and providing more effective and comfortable solutions for patients.^130^

###  Microemulsions (MEs)

 MEs are stabilized, transparent (or translucent) dispersions of two immiscible liquids (water and oil). They are spontaneously formed with a mean droplet diameter ranging from 0.01 to 0.14 µm, containing the surfactant/cosurfactant film as a boundary between the aqueous and oily phases. MEs are considered liquid membrane carriers to transfer either hydrophilic drug molecules through the aqueous media or lipophilic molecules across the lipidic phase.^131,132^ They have been used to increase the depth and transfer rate of moisturizing agents into the skin. It has been reported that MEs may disrupt the regulated-organized structure of the SC lipids, resulting in the loss of the skin’s outermost barrier properties and facilitating drug delivery to deep layers.^133,134^

 Several studies assessed the ME formulation of some whitening compounds or drugs useful in hyperpigmentation disorders. These studies evaluated the drug ME on cells to look for any enhancement in skin permeation, stability, and toxicity. From all we know, we did not find any accepted clinical trial in journals evaluating the effect of topical ME on hyperpigmentation or melasma. However, this drug formulation has great potential and can be a candidate for future studies. Tsai et al^135^ demonstrated that encapsulation in MEs could enhance *in-vitro* skin permeation of topical hesperetin. Hesperetin is a flavonoid compound with anti-inflammatory and antioxidant. In addition, topical hesperetin has a whitening effect. Topical delivery of hesperetin faced some permeation issues; therefore, MEformulation is used. Moreover, the efficacy of hesperetin-loaded MEs as a topical formulation for hyper-melanotic disease treatment was also illustrated, along with superior skin whitening effects and a depigmenting effect, as well as less irritation compared to the control group.

 In another similar study by Lin et al^136^ ascorbic acid 2-glucoside (AA2G), which has a whitening effect, was encapsulated in MEs to improve the skin diffusivity and stability compared to the solution form. In addition, studies have revealed that micro emulsifying of HQ could significantly improve its stability against photodegradation and increase dispersion over the SC.^137,138^ These MEs had 99.9% HQ content and remained visibly apparent during six months of storage, protecting the drug without antioxidants.^138^ In this context, any modifications in the composition and content of MEs can alter the permeability parameters and physicochemical properties.^137,138^

 Research conducted by Chang et al^139^ evaluated the cosmetic ME encapsulated deoxy arbutin (DA). As mentioned in the article, DA is a tyrosinase inhibitor with skin-lightening activity and no apparent cytotoxicity. Researchers developed oil-in-water (O/W) MEs with polysorbate series surfactant. MEs were stable at different storage periods. The anti-melanogenesis activity of MEs containing DA on B16-F10 mouse melanoma cells was better than that of the free DA. Brathwaite et al^140^ were other researchers who studied depigmenting activity of *Pouteria macrophylla* fruit extract ME on pigmented skin model. *Pouteria macrophylla* fruit extract, with the presence of gallic acid and other components, presented a cosmetic interest due to its anti-melanogenic action by inhibiting tyrosinase. The developed MEs were comprised of chromophore EL/span 80, ethyl oleate and HEPES (2-[4-(2-hydroxyethyl)-piperazin-1-yl]-ethanesulfonic acid). The tyrosinase inhibition of extract-loaded ME was twice better than conventional emulsion.

###  Nanoemulsions (NEs)

 NEs are thermodynamically stable oil in water dispersions with an average diameter of 20 to 200 nm. NEs are helpful for various drug delivery applications, particularly cutaneous and transdermal, and have the advantages of low toxicity, no irritation, and long-term stability.^141^ Due to their ability to improve different medications’ penetration across various skin layers and target poorly soluble pharmaceuticals, NEs are considered promising topical drug delivery systems. NEs effectively promote skin penetration and prolong drug retention time at the target site, reducing side effects.^142^

 Several studies assessed the NE formulation of whitening agents and drugs for hyperpigmentation. The studies used cells to evaluate the drug’s effects on skin permeation, stability, and toxicity. However, there is only one clinical trial evaluating topical NE’s effect on hyperpigmentation, not melasma, and it is not generally publicized. Räsänen et al^143^ investigated the efficacy of photodynamic therapy using aminolaevulinic acid NE as a light-sensitizing cream for lentigo maligna (an in-situ form of melanoma). The result of this interventional study is not officially reported. Even so, this drug formulation has great potential and can be a candidate for future studies on melasma.

 Jacobus Berlitz et al^144^ developed a double targeting strategy by azelaic acid-loaded NEs with HA to improve drug deposition and tyrosinase inhibitory effect. In this study, cytotoxicity, permeability, and mushroom tyrosinase inhibition assays were all performed *in-vitro*. According to the results, NEs significantly reduced tyrosinase activity penetrated the skin and reached the viable EP and dermis without cytotoxicity. Furthermore, the value of IC50 for the free form of azelaic was measured to be about 50 mM, while the NEs form represented an IC50 value of about 13 mM. In another study, kojic monooleate (the esterified form of KA) was successfully encapsulated within NEs to improve skin diffusivity. The IC50 value of NEs did not reach the maximal concentration of 100 µg/mL compared to the free form, *in-vitro*. Accordingly, the less cytotoxic formulation suggests excellent safety and may be used for cosmeceutical purposes.^145^

 Research conducted by Silva et al^146^ assessed the antioxidant and anti-tyrosinase activities of quercetin-loaded olive oil NEs on skin hyperpigmentation. Quercetin and olive oil are potential treatments for hyperpigmentation because of their antioxidant activity and anti-tyrosinase property. However, quercetin and olive oil have low water solubility and high chemical instability, which limits their application. NEs would overcome these limitations along with providing better antioxidant, anti-tyrosinase activity and less *in-vitro* toxicity. Zilles et al^147^ studied kojic dipalmitate NE formulation for treating melasma. Kojic dipalmitate, the esterified form of kojic acid, and rosehip oil have antioxidant and skin-regenerating properties. NE contained kojic dipalmitate, rosehip oil and surfactant and were made by the Ultra-Turrax method. Kojic dipalmitate-containing NEs showed better antioxidant and depigmenting efficacy with no cytotoxicity on porcine ear cells.

 In the study performed by Azimuddin et al,^148^ the NE formulation of kojic monooleate on hyperpigmentation was studied. Kojic monooleate is an ester form of kojic acid and oleic acid with a better depigmenting effect than kojic acid. Kojic monooleate-containing NE on mouse embryonic fibroblast cells showed better tyrosinase inhibition and no cytotoxicity. In another research, Azimuddin et al^149^ evaluated nanoemulsified kojic monooleate for cosmeceutical application. With a molecular docking stimulation, kojic monooleate was characterized and optimized. Researchers further assessed the application of kojic monooleate NE for skin hyperpigmentation. Another research conducted by Tang et al^150^ studied topical delivery and anti-melanogenic effects of lecithin-based NE tetrahydro curcumin (THC). THC is known for its antioxidant properties, especially for stress-induced diseases like skin hyperpigmentation. Based on Tang study reports, NE form of THC, significantly enhanced the membrane permeation compared to the free form of THC. NEs increased the anti-melanogenic effect of THC. Budama-Kilinc et al^151^ aimed to develop a chlorogenic acid NE formulation for hyperpigmentation disorder. Chlorogenic acid (CA) is an ester of L-quinic acid and caffeic acid. It is known for its anti-inflammatory, anticancer activities, and protective role in oxidative stress-related diseases like hyperpigmentation. Chlorogenic acid NE has better stability and permeation than free chlorogenic acid. In addition, enhanced anti-tyrosinase, anti-melanogenesis, and decreased toxicity in the melanoma B16F0 cell line have been seen.

 In another study, Ting et al^152^ evaluated nano emulsified adlay bran oil (ABO) anti-tyrosinase activity and melanin synthesis inhibition. ABO, a traditional anti-inflammatory medicine in Asia, inhibits melanin biosynthesis. Nevertheless, oily components like ABO are often hard to evaluate because of their incompatibility. ABO-loaded NE showed a dose-dependent inhibition in tyrosinase activity and melanin production with *in-vitro* (mouse melanoma B16F10 Cell) and *in-vivo* (zebrafish embryos) systems. Researchers concluded that ABO- loaded NEhas excellent potential as an anti-hyper pigmenting agent. Tofani et al^153^ conducted an *in-vitro* diffusion study to compare NLC and NEforms of deoxy arbutin (DA). As mentioned, DA has anti-hyperpigmentation effects and is a better tyrosinase inhibitor than HQ. *In-vitro* penetration of NLC, NE, and conventional emulsion form of DA was evaluated. As researchers concluded, the NLC form showed better topical delivery and improved depigmenting efficacy than other forms.

###  Lipid nanoparticles (LNPs)

 LNPs are colloidal drug delivery systems that exploit various mixtures of surfactants and cosurfactant for stabilization. Typically, LNPs include NLCs and SLNs. As a new generation of lipidic nanocarriers for drug delivery, NLCs are produced by integrating the core matrix of an oil-in-water emulsion with a mixture of solid and liquid lipids. SLNs comprise lipid components in the solid state at room temperature and form the core matrix with particle sizes ranging from 50 to 1000 nm.^154,155^

 Since lipid-based drug delivery systems have small particle sizes and are in close contact with the SC, they can enhance the dermal penetration of drugs. Moreover, they can provide a thick layer on the skin surface that possess occlusive effects and improves skin hydration. Using surfactants in their formulation, which can fluidize or loosen the SC structure, contributes to increased skin permeability. Medications are first released from the nanoparticles during skin penetration, then partitioned to different cells and absorbed.^156,157^ Several studies evaluated the NLC and SLN formulation of whitening compounds and drugs for hyperpigmentation disorders. However, from all we know, we were still looking for an interventional study, specifically on melasma. Aceto et al^158^ investigated the effect of NLC and SLN formulation of ginger oil (GO) and hexylresorcinol on hyperpigmentation in a double-blind, single-center and *in-vitro* study is investigated. Hexylresorcinol (HR), a tyrosine enzyme alternative substrate, outclasses tyrosinase-related protein affinity to tyrosine. GO is an oily extract inhibiting MITF expression and tyrosinase activity. By LNPformulations application, dual delivery of HR and GO is possible. One hundred healthy Caucasian women were randomized into the active treatment group (NLC and SLN) and inactive control group twice daily for 28 days. SLNSs and NLCs showed decreased skin pigmentation and augmented skin lightness compared to the control group. HR and GO were successfully encapsulated within NLC and SLN and showed an enhanced tyrosinase inhibition effect. Other researchers tested the drug with SLN or NLC on cells or animals to assess skin permeation, stability, and toxicity. LNP drug formulation has great potential and can be a candidate for future studies.

####  Solid lipid nanoparticle (SLN)

 Despite the hydrophilic nature of molecules, SLNs were demonstrated to entrap HQ effectively. Although these HQ-loaded nanoparticles revealed greater skin penetration and higher stability against oxidation. Compared to topical HQ hydrogel, HQ-loaded SLN hydrogel had a nearly 3-fold higher drug accumulation in the skin, *in-vitro*. These findings indicated that HQ-loaded SLNs had reduced systemic absorption and better skin localization. Accordingly, due to the biphasic nature of these nanoparticles, excellent stability against oxidation, good skin penetration, low systemic absorption, and adequate loading value, SLNs are found to be valuable colloidal drug delivery carriers for topical administration of HQ in the treatment of hyperpigmentation.^159,160^ Ghanbarzadeh et al^159^ aimed to study the stability of HQ and dermal delivery with SLNs. The researchers found that HQ limitations like instability, insufficient skin penetration (because of hydrophilic structure), and severe side effects can be overcome by SLN formulation. Some other researchers, Talele et al,^160^ formulated HQ in SLNs comprised of stearic acid and ionic emulsifiers. Like other studies, HQ-loaded SLNs helped overcome the drawbacks of free HQ.

 The therapeutic use of topical curcumin (Cur) in treating hyperpigmentation is restricted due to its low solubility. In this context, the potential application of LNPs to enhance the solubility of drugs is drawing interest. Shrotriya et al^161^ developed a topical Cur encapsulated SLN (Cur-SLN) gel and evaluated its tyrosinase inhibitory activity. Their findings showed that the therapeutic function of Cur was significantly elevated by incorporation into SLNs, compared to reference Cur-plain gel. Consequently, including lipids in skin whitening products may promote the development of these formulations since lipids are more easily absorbed by cells.^161^ In another study by Dzulhi et al,^162^ the green tea (*Camellia sinensis* L.) leaf extract, which contains high levels of polyphenolic compounds with anti-tyrosinase properties, was successfully loaded into SLNs. Marto et al^163^ formulated an SLN form of N-acetyl-D-glucosamine (NAG). NAG, a precursor of HAhas been considered for anti-hyperpigmentation agent due to its inhibitory effect on tyrosinase enzymes. Oil emulgel and hydrogel were used as carriers of NAG-loaded SLN. The researchers concluded improved topical delivery and better treatment for skin disorders with NAG-loaded SLN than free NAG. In the study by Kabiri et al,^164^ SLN formulation Udecylenoyl phenylalanine (UP) is made to investigate anti-tyrosinase activity. UP, a commercial lipophilic derivate of phenylalanine, is a new brightener for skin pigmentation disorders. As a hydrophobic drug with some limitations on its efficiency, an SLN formulation is considered. The *in-vitro* permeation experiment revealed enhanced cutaneous uptake and melanogenesis inhibition with anti-tyrosinase activity for UP-loaded SLN. Khezri et al^165^ performed an *in-vitro* study evaluating the treatment of hyperpigmentation disorders with kojic acid-SLN. Kojic acid is a tyrosinase inhibitor with insufficient skin penetration and several adverse effects. Loading kojic acid in an SLN formulation is followed by more tyrosinase inhibition potency and percutaneous delivery than pure kojic acid.

 Other researchers, AL-Amin et al,^165^ investigated the therapeutic efficacy of MHY498 SLNformulation for treating skin hyperpigmentation. MHY498 is a newly synthesized potent tyrosinase inhibitor containing imidazole and 2,4-dihydroxy phenyl moiety. MHY498-loaded SLNs were prepared by an oil-in-water emulsion solvent-evaporation method. MHY- SLN had a prolonged drug-release profile and higher skin permeation than the MHY solution. Also, this formulation showed a prominent inhibitory effect against UV B-induced melanogenesis in C57BL/6 mice. Mostafa et al^166^ developed an SLN form of ethanol leaves extract of *Prunus persica* (PPEE) for skin whitening effects. *Prunus persica* leaves contain phenolic and flavonoid content with notable antioxidant activity. *In-vitro* investigation of PPEE-SLN on human keratinocyte cell lines showed higher tyrosinase inhibitory effects. In addition, a protective effect against UV on mice was observed.

###  Nanostructured lipid carrier (NLC)

 Several compounds with anti-tyrosinase activity, including plant-derived extracts, have been formulated with LNPs. Due to high levels of trans-resveratrol and its derivatives, melinjo (*Gnetum gnemon* L.) seed extract (MSE) has shown potential skin-whitening properties by blocking tyrosinase activity. Therefore, MSE can apply against the melanogenesis process. Ayuningtyas et al^167^ examined the therapeutic benefits and negative consequences of employing a MSE-loaded SLNs. Accordingly, the results did not cause skin irritation. Moreover, after 28 days of application, the skin MIwas significantly decreased (*P* value < 0.05), on average. Tofani et al^153^ studied different lipid structures for topical delivery of DA with potential depigmenting properties. DA is a tetrahydropyranyloxy phenol that acts as a relatively new skin-lightening agent with higher tyrosinase inhibitory effects than HQ and less cytotoxicity against melanocytes and other cells. The *in-vitro* penetration study was performed using Spangler’s membrane, comparing the efficacy of different delivery techniques, including NE-loaded gel, conventional cream, and NLC-loaded gel. After 8 hours of follow-up, DA-loaded NLC gel demonstrated better penetration than the others.^153^

 Phenylethyl resorcinol (PR) is an antioxidant active ingredient and skin-lightening agent naturally derived from pine trees. It has been demonstrated that loading PR into NLCs can increase the drug’s solubility, photostability, and anti-tyrosinase properties against melanoma cells. NLCs could effectively transfer PR into deep layers of skin and melanoma cells, according to the results from a cellular uptake study and tyrosinase inhibition assay on B16F10 cells.^168^

**Figure 8:**
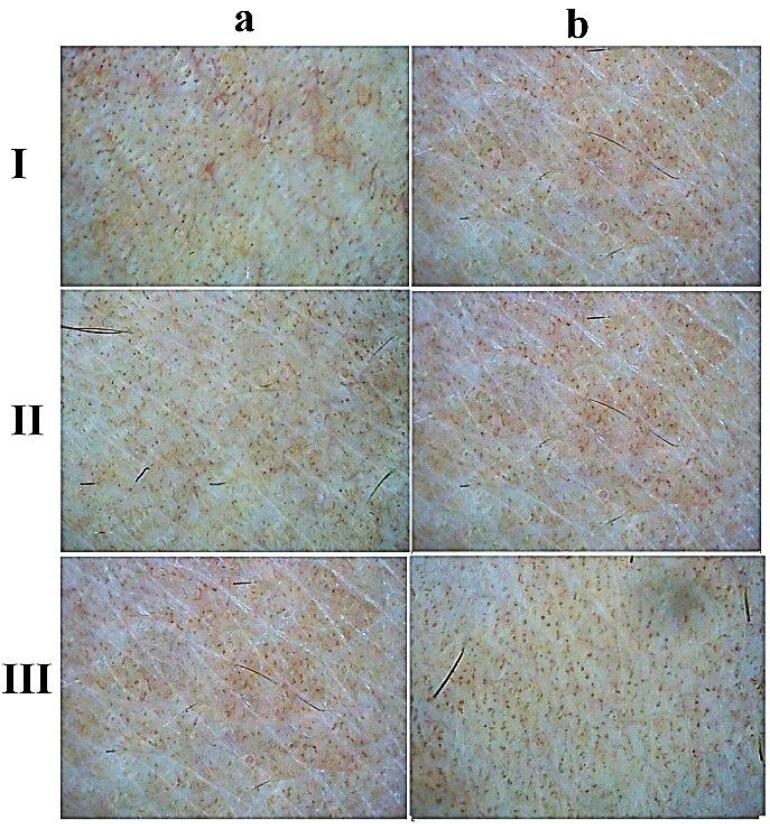
Polarized dermoscopy images (a) before treatment, (b) after 8 weeks of treatment with N-acetyl-glucoamine -loaded nanostructured lipid carriers (I), blank nanostructured lipid carriers (II), and N-acetyl-glucoamine solution (III)^169^

 N-acetyl-glucosamine (NAGA) is an amide derivative of the monosaccharide glucose that can significantly inhibit melanin production and alter the expression of several pigment-related genes. Despite these advantages, NAGA cannot be used in topical formulations due to its high polar characteristics and remarkable hydrophilicity. A brief clinical study involving three groups of participants was conducted to support this assertion. The first group received the NAGA-loaded NLCsformulation every night to the right inferior forearm (group I, test group). The left forearm of the second group (group II, negative control) received blank NLC (placebo) every night. Finally, to determine whether NAGA-loaded NLCs exhibit a superior lightening effect than NAGAconventional solution, a NAGA solution was administered to the third group (group III, positive control) every night. Following an eight-week treatment period, no notable variation in skin melanin density was observed between the blank NLCs and NAGA solution. However, dermoscopy images showed a significant reduction in the pattern of melanin distribution in most cases treated with NAGA-loaded NLCs^169^ (Figure 8). This fact revealed that the penetration of highly polar compounds such as NAGA into SC is more challenging, while small-sized NLC particles can penetrate easily.^170^

 In a study by Wu et al,^171^ HQ was loaded in NLCs. Their results showed higher stability, increased permeability, improved drug distribution, and diminished skin irritation. Finally, NLC is concluded as a potential delivery vehicle for transdermal products in the future. Also, Sharifmakhmalzadeh et al^172^ evaluated the effect of HQ-loaded NLCs on rat skin. As researchers concluded, the HQ-loaded NLC formulation has favorable physicochemical properties, including particle size, release behavior, entrapment efficiency percentage, HQ permeation amount through rat skin after 4 and 28 hours, and stability for topical use. Banna et al^173^ conducted research on MHY908-NLC for treating hyperpigmentation. MHY908 is a newly synthesized tyrosinase inhibitor with greater binding affinity than kojic acid. MHY-NLCs^6^ exhibited superior skin permeation and a dual drug release profile compared to the MHY908 solution. The drug exhibited an initial burst release in both *in-vitro* and *ex-vivo* studies, followed by a more pronounced occlusion effect, resulting in a prolonged drug release.

 The anti-tyrosinase activity of trans-resveratrol with NLC carrier was evaluated by Fachinetti et al^174^ Trans-resveratrol (RSV) is a natural compound that inhibits the tyrosinase enzyme and skin-lightning effect. The NLC carrier was made of glyceryl behenate or polyoxymethylene 40 stearate. RSV-loaded NLCs showed enhanced tyrosinase inhibition and less L-929 cell compared. In addition, PEG 40 stearate NLC presented smaller particle sizes and better emulsification and nanoparticle formation than glyceryl behenate NLC. Danehmand et al^175^ loaded auraptene (AUR) into NLCs and investigated its melanogenesis inhibitory activities. AUR, a natural antioxidant agent, can treat hyperpigmentation. AUR-NLC has prolonged drug release, increased tyrosinase inhibition, and less cytotoxicity on the B16F10 cell line.

###  Liposomes

 Liposomes are small microscopic vesicular structures with an exterior lipid bilayer spherical membrane providing hydrophobic and hydrophilic environments. Liposomes may involve a variety of components, the most important of which are phospholipids and cholesterol.^176^ Scientifically, liposomes are divided into small unilamellar vesicles (SUVs), large unilamellar vesicles (LUVs), and large multilamellar vesicles (MLVs) or multivesicular vesicles (MVVs), which has a particle size of about 100 nm, 200 to 800 nm, and 500 up to 5000 nm, respectively.^177^

 Liposomes with an average diameter of 300 nm can penetrate deep layers of the skin. An average diameter of about 70 nm is optimal for dermal delivery of particles. However, liposomes with an average diameter of 600 nm and more cannot penetrate deep skin layers and mostly remain on the surface of the SC.^178^ In topical administrations, liposomes provide several advantages as vesicular nanocarriers, including regulated drug release, enhanced absorption, localized drug deposition in skin layers, decreased systemic absorption, and fewer adverse effects. Studies have demonstrated reduced serum levels and urine excretion of the drug molecules with liposome-containing formulations, indicating localized skin deposition.^179^ Potential applications for transdermal delivery of medications are another pinpoint toward liposomal usage. Studies have shown that new deformable vesicles called transferosomes are preferable to traditional liposomes in this context. Transferosomes are vesicular self-aggregating vesicles with highly flexible membranes that can deliver drugs into or through the skin.^180-182^

 Surfactants can increase the flexibility and stretchiness of liposomes’ lipid bilayer structure, dramatically promoting drug penetration through EP and transdermal drug delivery.^182^ Further, adding ethanol to liposome composition, ethosome, may also improve vesicle flexibility and enhance absorption efficacy.^183^ Paolino et al^180^ performed an *in-vitro* and *in-vivo* research which demonstrated that flexible and elastic vesicular nanoparticles are preferred over stiff liposomes for topical delivery to improve skin penetration.

 Results of the skin accumulation studies for the PR penetration model have shown that elastic formulations (such as transferosomes) have superior drug retention in the skin, higher tyrosinase inhibitory activity and reduced melanin content compared to conventional liposomes in B16 melanoma cells. Therefore, these elastic vesicular carriers provided a more stable delivery system for PR that is highly effective as skin lightening product.^181^ The potential applications of linoleic acid-loaded transferosomes and ethosomes as topical delivery systems in the management of melasma or hyperpigmentation disorders have been studied by Celia et al.^184^ Accordingly, nanocarriers can increase the stability of linoleic acid and enhance deep skin penetration through different layers. These findings highlight the efficacy of these nanocarriers in the treatment of melasma. Scientifically, the superior permeability of transferosomes contributes to the highly deformable nature of these particles, while ethosome-associated enhanced permeability is due to the inclusion of ethanol in their formulation.^184^ In another study, Lee et al^185^ developed topical transferosomes containing niacinamide (NA) to treat hyperpigmentation disorders. Compared to traditional liposomes, these particles showed improved whitening properties, skin permeability, and superior capability to entrap molecules due to their flexibility.^185^ The therapeutic effects of HQ-loaded liposomes against melasma and hyperpigmented patches were explored by Taghavi et al.^186^ They found that HQ-loaded liposomes presented significantly higher stability over the regular HQ cream. Throughout the study, there was no noteworthy contrast in the MASI score between the test and control groups. Banihashemi et al^187^ compared the therapeutic effects of TXA-loaded liposomal cream over the conventional HQ cream on treating hyperpigmented patches and melasma. A split-face study that enrolled thirty women with bilateral melasma was conducted. During the 12 weeks of follow-up, participants randomly received 4% HQ cream and 5% liposomal TXA cream on different sides of their faces. At the end of the follow-up, there was no discernible change in the average MASI scores. However, the liposomal TXA-treated side did not show any side effects, while the HQ-treated side experienced irritation.^187^

 The hydroglycolic extract from *Artocarpus lakoocha* (AL) heartwood contains high amounts of phenolic and flavonoid components associated with anti-inflammatory and anti-tyrosinase properties. Teeranachaideekul et al^188^ explored the efficacy of AL heartwood extract in both liposomal and non-liposomal (free) forms for skin-whining and treating melasma. The volunteer’s skin was observed to be whether whiter or lighter after applying these two forms. According to the results, the liposomal form of AL heartwood extract exhibited better whitening efficacy. In another study, liposomes were exploited to increase the topical bioavailability of *Aloe vera* leaf gel extract (AGE). These liposomes had a high encapsulation efficiency for incorporating AGE. Two groups of 90 pregnant women suffering from melasma were enrolled on this double-blinded, randomized clinical trial. Participants in the test group received liposome-encapsulated AGE (in the form of a gel), while the control group received an AGE-free form. After five weeks of beginning the melasma treatment, the liposomal-AGE group showed a 32% improvement in the MASI score compared to a 10% improvement in the AGE group alone. Moreover, at the end of the follow-up, the average MASI score showed a significant difference between the test and control groups.^189^ Huh et al^190^ also discussed the idea of encapsulating 4-n-butyl resorcinol (4Nbr), a resorcinol derivative with a melanogenesis inhibitory effect, in liposomes to increase the molecular stability, skin penetration, and tyrosinase inhibitory properties. After eight weeks of treatment with 0.1% 4Nbr liposomal cream, more than 60% of patients presented melasma improvement without any side effects. Aside from that, the efficacy and tolerability of liposomal 4Nbr cream combined with resveratrol to prevent melanogenesis has also been studied by Kwon et al.^191^ During four weeks of treatment, 21 women with melasma were treated. According to their results, this formulation was remarkably effective and safe. The positive effects may appear as early as two weeks.

 The study examined the effectiveness and safety of using a liposomal form of azelaic acid 20% in conjunction with oral TXA to treat melasma, compared to conventional 4% HQ cream. Two equal groups of twenty-five female participants with melasma diagnosis were enrolled. The control group was treated with HQ 4%, while the test group received the liposomal form of azelaic acid 20%. Both groups received 250 mg of TXA as a daily oral dose. The outcome of this study revealed that females who received the liposomal form of azelaic acid experienced better melasma improvement and significant positive effects. As regards the test group, the MASI score decreased from 17.06 ± 1.51 to 5.58 ± 1.28, whereas in the control group, it decreased from. Furthermore, the liposomal form of azelaic acid was dramatically more tolerable than HQ 4%.^192^ Gamea et al^193^ compared liposomal TXA and its combination with PRP in an interventional study. Dermatologists and plastic surgeons often utilize PRP to treat chronic wounds, ulcers, and acne scars. This study was conducted with forty female patients who had melasma and were randomly divided into two groups. Both groups received a topical liposomal-TXA 5% treatment on both sides of their face. One group also received an intradermal injection of topical liposomal TXA 5% plus PRP for four sessions at three-week intervals. After treatment sessions, the mMASI score decreased on average by 8.5 for PRP added group and 3.9 for another group (*P* < 0.05). Liposomal TXA 5% was safe and effective for the treatment of melasma.

**Figure 9:**
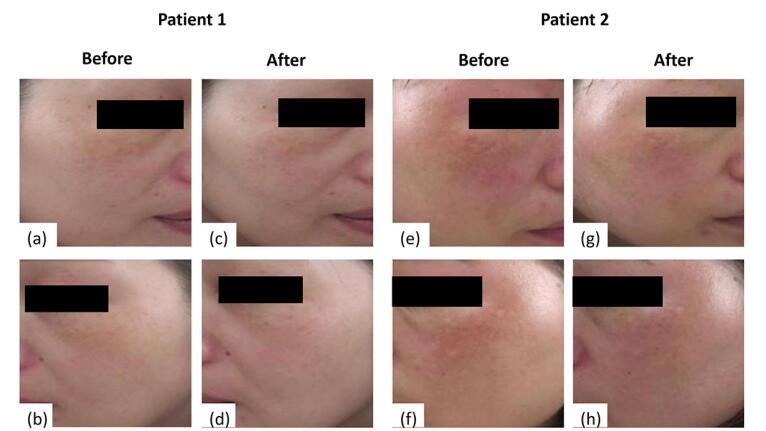
Photographs (a, b, e, f) represents before patients’ treatment and (c, d, g, h) shows Improvement of melasma patients after three-month use of topical 1.8% tranexamic acid liposome. Reprinted with permission from Xing et al^48^

 Liposomal Politranexamide was evaluated on melasma in a prospective, randomize, single-blind study by Manfreda et al.^194^ Politranexamide consist of a liposomal emulsion of TXA. Twenty-six patients with facial melasma were divided into two groups receiving Politranexamide or a competitor based on acetylglucosamine, ethyl linoleate, and phenyl ethyl resorcinol twice daily for 12 weeks. MASI score was reduced significantly for both groups suggesting Politranexamide as a valuable and safe therapeutic option in treating melasma. Xing et al^48^ studied the efficacy and safety of liposomal or MN formof TXA to treat melasma (Figure 9). Sixty patients with melasma included the study to receive either topical liposomal TXA 1.8% twice a day or TXA 5% solution MN weekly or conventional HQ 2% every night. 27.8% of patients using liposomal form and 33.3 of patients using MNshowed 50% improvement at the end of treatment. Also, a significant decrease in the MIwas seen. Xing et al^48^ study suggest both liposome and MNformof TXA as a promising option for treating melasma.

###  Niosomes

 Niosomes are new drug delivery systems introduced as an alternative to traditional liposomes. They are vesicular microscopic lipid bilayer structures formed by combining non-ionic surfactants, cholesterol, and charge-inducing agents. Fundamentally, non-ionic surfactants are biocompatible and non-toxic. They can improve the topical penetration of different drugs. Surfactants with high HLB values cannot form vesicles due to their high hydrophilicity, although Spans and Tweens are excellent non-ionic surfactants for noisome preparation.^195,196^ Niosomes may alert the compact lipid structure of the SC, making it looser and more permeable. The primary mechanisms suggested for this proposal are causing a significant reduction in trans-epidermal water loss. Fusion or absorption of these vesicular drug delivery systems to the skin surface is another mechanism for increased permeability, resulting in a higher concentration gradient and skin penetration.^197^ Ammar et al^198^ developed HQ niosomal gel as a topical drug delivery system to enhance depigmentation effects for the treatment of melasma. The test group received 2% HQ niosomal gel in this trial, whereas the control group were treated with conventional HQ cream every night. At the end of the follow-up, the two groups significantly differed in the clinical response, adverse effects, recovery duration, melasma recurrence, and modified MASI score. In this context, the recurrence rate of melasma in the control group was more than twice that of the niosomal gel group.^198^

 Due to its anti-tyrosinase activity, a topical form of NAG has recently been used to treat hyperpigmentation disorders. Shatalebi et al^199^ studied the skin penetration of NAG niosomes through rat skin using a Franz diffusion cell. Accordingly, niosomal formulations significantly improved drug permeability across the skin compared to a NAG-loaded HA solution. Moreover, the outcomes of this study indicated that niosomes have the potential to improve NAGlocalization in the skin, which is required in hyperpigmentation diseases.^199^ Similarly, potential applications of PRP-loaded niosomes for improved skin whitening and enhanced stability and skin permeability were demonstrated.^200^ In another study by Desnita et al,^201^ alpha arbutin was successfully encapsulated into niosomes. Based on their results, niosomal formulations presented higher permeability and whitening efficacy *in-vitro*. Kandil *et al*^202^ investigated magnesium ascorbyl phosphate (MAP) niosomal and ethosomal form, *in-vitro*, *ex-vivo,* and in a clinical study. MAP is a stabilized ascorbic acid derivative that has shown enhanced stability in cosmetic formulation. In an *in-vitro* study, ethosome and niosome forms of MAP were optimized and incorporated into Carbopol gel. Optimized ethosome and niosomes showed comparable controlled permeation, higher skin retention, and improved stability in an *ex-vivo* study on rat skin. Finally, 40 patients with acquired facial hyperpigmentation and melasma were given niosomal gel MAP 5% for the right side of the face and ethosomal gel MAP 5% for the left side once at night for six months. After one month, ethosomal MAP showed a significant decrease in MI, while niosomal MAP showed treatment improvement after six months. Kandil et al^202^ concluded, combining ethosome and niosome forms of MAP could be promising skincare formulations for melasma and hyperpigmentation short and long-term treatment.

 Arbutin niosomes, called arbusomes, were investigated for treating hyperpigmentation in an *in-vitro* and *ex-vivo* study by Radmand et al.^203^ Arbutin is a HQ D-glucopyranoside derivative with skin-lightening effects. Arbusome showed better skin permeation and less cell cytotoxicity on the HFF cell line than simple arbutin. There was no irritation for arbusome on the skin of Wistar rats. In another study, Saeedi et al^204^ developed kojisome, a noisome form of kojic acid, and evaluated its anti-melanogenesis and antioxidant effects. Kojisome permeation enhancement is revealed because of increased kojic acid in dermal layers than of simple kojic acid. Kojisome exhibited high safety profile and low cytotoxicity. Also, kojisome inhibited melanin formulation more than free kojic acid.

###  Invasomes

 Invasomes are an emerging class of vesicular lipid-based systems with remarkable skin penetration properties over traditional liposomes. Skin penetration-enhancing properties of invasomes are attributed to their capacity to damage SC lipids and interact with intracellular proteins, which increases drug partitionation in the SC. They primarily consist of ethanol, phospholipids (phosphatidylcholine), and terpenes. As natural volatile oils, terpenes are considered benign penetration enhancers with restricted irritation potential at low doses (1-5%). Further, their reversible impact on SC lipids makes them therapeutically more acceptable.^205,206^ Therefore, potential applications of invasome as topical drug delivery systems for melasma treatment have been discussed. These vesicular particles can possess significant therapeutic effects due to their high deformability. Moreover, invasomes provided deeper skin penetration of PR than the transfersomes and conventional liposomes, which attribute to their high ethanolic and terpene content.^181^

###  Ethosomes

 Ethosome is a carrier for transdermal drug delivery to increase the amount of drug permeation through the skin. They have been found to allow hydrophilic drugs to cross the SCbarrier and ultimately improve the bioavailability of the drug. Therefore, ethosome-based formulations have been widely used in a variety of skin pathologies.^207^

 These drug carriers are phospholipid-based elastic nanovesicles containing a high content of ethanol. Also, ethanol, an efficient permeation enhancer, can interact with the lipid molecules’ polar head group region, reducing the SClipid’s melting point, thereby increasing lipid fluidity and cell membrane permeability. The high flexibility of vesicular membranes from the added ethanol permits the elastic vesicles to squeeze themselves through the pores much smaller than their diameters.^208^ Guo et al^207^ investigated the effect of ethosomal form of TXA on melasma. Eighty-eight participants with melasma enrolled in a double-blind, placebo-controlled, randomized, prospective study. Patients were randomized 1:1 to two groups, moisturizing mask with ethosomal-TXA 0.5% and moisturizing mask only, once daily for two weeks and once every other day for the third and fourth weeks. Reduction in vascular score measured at the end of treatment was significantly greater than the control group, suggesting Ethosome-TXA as an effective treatment for melasma. However, due to the high concentration of volatile ethanol in ethosomes, there may be a problem with ethosome-induced skin irritation, and researchers shortened the duration of treatment to 1 month to avoid potential adverse effects. In another study, kojic acid dipalmitate was loaded into ethosomal gel for treating hyperpigmentation. Tanveer et al^209^ conducted an *in-vitro* study to optimize the ethosome formulation and an *in-vivo* study on animal to the investigate the anti-hyperpigmentation activity. Kojic acid dipalmitate-loaded ethosomes founded to be stable and offers a promising therapeutic approach for skin whitening and moisturizing.

###  Polymertic nanoparticles

 In recent years, topical polymeric-based drug delivery systems drawn interest in treating several dermatological conditions due to their controlled-release properties and promoted drug dosition inside the skin. Chitosan-based nanoparticles are among this contest’s most promising topical drug delivery systems in this context. The N-deacetylated chitin derivative known as chitosan mainly compreses D-glucosamine and NAG units. As a natural cationic polymer, chitosan is biocompatible, non-toxic, and biodegradable. It has been found to have various advantages for topical applications due to its high positive charge, penetration-enhancing potential, and skin protection properties against environmentally destructive factors such as the sun.^210,211^ Hatem et al^212^ investigated dermal delivery of chitosan-based nanoparticles loaded with arbutin in the gel form as skin-whitening agents for enhanced treatment of melasma. Chitosan-based nanoparticles were primarily functionalized with HA and collagen derivates using the ionic gelation approach for this proposal. Different physical and chemical analyses (such as FT-IR analysis and differential scanning calorimetry) and drug encapsulation efficacy were performed to ensure the successful preparation of nanoparticles. According to the results, these chitosan-based nanoparticles were positively charged, spherical, and stable during three months of follow-up. They also demonstrated an enhanced sustained release profile of arbutin over 24 hours. In addition, an improved ability to deliver the therapeutic molecules directly into the deeper layers of the skin and superior clinical outcomes were observed. These results may highlight nanoparticulated systems’ critical role in overcoming dermatological disorders, including melasma treatment.^212^

 Recently, topical TXA has been considered an emerging melasma treatment option. Nassar et al^213^ conducted a comparative study of the therapeutic effects of Kligman’s conventional triple therapy and topical 3% TXA-loaded chitosan mircoparticles (CsMPs) gel for treating melasma. Twenty-four female participants with melsma enrolled on this randomized study. Participants were further divided into two equal groups based on the treatment regimen. One group received the conventional triole therapy once daily at night, while the other group were treated with topical 3% TXA-loaded CsMPsgel twice daily. According to the MASI score, participants were clinically assessed once a week for six weeks. Further, they were followed up for an additional three months after the end of treatment. In both groups, the MASI score was significantly lower than the baseline level (*P* < 0.05). However, the TXA-loaded CsMPsregimen provides higher efficacy and safety. In contrast, side effects like redness, irritation, and burning were more associated with the topical Kligman formula. No statistically significant difference was found between the two treatment regimens regarding patient compliance, treatment success, complications, and melasma recurrence.^213^ Lee et al^214^ found that chitosan liposomes enhanced the skin’s absorption of niacinamide. Cationic liposomes were applied twice daily to the left and right sides of the face for 28 days. The results showed significant improvement in skin brightness, MI, and melasma area where cationic liposomes were used compared to the control formulation without cationic liposomes.

 In a research, Hatem et al^212^ used chitosan-based nanoparticles form of skin whitening agents like alpha-arbutin, HA, and collagen for melasma treatment. Researchers discovered that chitosan nanoparticles can deliver drugs into deeper layers of the skin without needing transdermal delivery. They also found that this method is clinically superior to conventional therapeutic forms when treating melasma, indicating that nanoparticulate systems could treat various dermatological disorders. Table 2 provides a overview of the various Nanocarrier-Based methods and their effects on melasma.

**Table 2:** Summery of nanoparticels with potential applications in the tretment of melasma.

**Type of nanoparticels**	**Content**	**Intervention**	**Sample size (nm)**	**Treatment duration**	**Treatment follow-up**	**Clinical outcome**	**Ref**
NLC	AGA	NLC with AGA, NLCalone	15	8 weeks	2 months	Dermatologists visually examined the test and control groups and found no significant differences. This result may be attributed to the small sample size, limited scoring system, or short treatment period.	^169^
SLNs and NLC	Hexylresorcinol, ginger oil	NLC with Hexylresorcinol, ginger oil / SLNs with Hexylresorcinol, ginger oil	96 to 119	28 days	4 weeks	The NLC ones proved to be more effective than SLN, as they resulted in higher percentages of MI change.	^158^
Liposomes	NA, Bounsphere^TM^	Niacinamide (NA), Topical transferosomes (Bounsphere^TM^),	21	8 weeks	6 months	Skin whitening efficacy confirmed that Bounsphere^TM^ suspension containing 2% NA improved melasma.	^185^
	HQ	Liposomal HQ (A), Conventional HQ (B)	20	12 weeks	3 months	(A):The mean MASI score decreased from 10.73 ± 4.7 to 6.25 ± 4.0 (B):The mean MASI score decreased from 10.73 ± 4.7 to 6.07 ± 3.8	^186^
	TXA, HQ	5% topical liposomal TXA, 4% HQ cream	23	every month	4 months	(A):The mean mMASI score decreased from 14.72 ± 2.2 to 6.78 ± 2.9 (B):The mean mMASI score decreased from 14.60 ± 2.3 to 7.60 ± 2.2	^187^
	Hydroglycolic	The hydroglycolic extract Artocarpus lakoocha heartwood	10	4 weeks	1 month	AL heartwood extract-loaded liposome lotion showed better skin-whitening effects than non-encapsulated extract lotion.	^188^
	Aloe vera	Liposomal-Aloe vera leaf gel extract (AGE) (A), AGE (B)	90	5 weeks	5 weeks	(A):The mean MASI score decreased from 15.0 ± 1.8 to 10.2 ± 2.0 (B):The mean MASI score decreased from 15.5 ± 2.4 to 13.9 ± 2.7	^189^
	4-n-butylresorcinol	Liposome-encapsulated 4-n-butylresorcinol 0.1% cream (A), liposome (vehicle) (B)	23	4 and 8 weeks	2 months	(A): the MI decreased from 200.68 ± 38.24 to 185.42 ± 38.81 (B): the MI decreased from 201.13 ± 39.78 to 194.43 ± 39.03	^190^
	4-n-butylresorcinol, resveratrol	Liposome 4‐n‐butylresorcinol (4nBR) + resveratrol (RSV)	21	2 and 4 weeks	1 month	-The MI decreased from 201.08 ± 25.76 to 182.83 ± 18.61 in lesional. -The MI decreased from 129.02 ± 21.54 to 128.32 ± 22.38 in nonlesional	^191^
	azelaic acid, TXA, HQ	Liposomal azelaic acid 20% + Oral TXA (A), HQ 4% + Oral TXA (B)	50	monthly interval	6 months	(A): the MASI score decreased from 17.06 ± 1.51 to 5.58 ± 1.28 (B): the MASI score decreased from 17.77 ± 1.45 to 7.18 ± 1.31	^192^
	TXA	Topical TXA 5% in a liposome(A), PRP (B)	40	(A): 12 weeks (B): every 3 weeks	1 month	(A): the MASI score decreased from 11.7 ± 2.98 to 7.8 ± 1.9 (B): the MASI score decreased from 12.1 ± 2.9 to 3.6 ± 1.9	^193^
	Politranexamide, acetylglucosamine, ethyl linoleate, and phenyl ethyl resorcinol	Liposomal emulsion based on Politranexamide (A), acetylglucosamine, ethyl linoleate, and phenyl ethyl resorcinol (B)	26	6 and 12 weeks	3 months	(A): the mean MASI score decreased from 10.9 ± 7.00 to 7.75 ± 5.22 (B): the mean MASI score decreased from 9.34 ± 6.29 to 7.61 ± 5.24	^194^
	TXA, HQ	1.8% liposomal TXA (A), MN with 5% TA solution (B), 2% HQ (C)	60	4, 8, and 12 weeks	3 months	(A): the MI decreased from 203.14 ± 10.27 to 178.33 ± 11.41 (B): the MI decreased from 261.13 ± 17.89 to 221.17 ± 18.83 (C): the MI decreased from 214.85 ± 10.19 to 176.48 ± 9.57	^48^
Niosome	HQ	Niosomal gel of HQ 2% in 2% carbopol (A), topical HQ 2% (B)	60	every 2 weeks	3 months	(A): the mean mMASI score decreased from 8.41 ± 5.82 to 1.09 ± 0.84 (B): the mean mMASI score decreased from 8.63 ± 6.09 to 3.36 ± 4.63	^198^
Ethosome	TXA	0.5% TXA (Test), moisturizing (Control)	88	4 weeks	4 months	The MASI score of the Test group decreased from 4.84 ± 2.08 to 4.47 ± 1.97 The MASI score of the Control group decreased from 4.93 ± 2.25 to 4.80 ± 2.00	^207^
Niosome, ethosome	MAP	MAP niosomal gel (A), MAP ethosomal gel (B)	40	6 weeks	6 months	(A): The mean melanin’s level decreased from 0.776 ± 0.1158 to 0.748 ± 0.1608 (B): The mean melanin’s level decreased from 0.792 ± 0.1321 to 0.758 ± 0.1619.	^202^
Polymeric nano parrticles (Chitosan)	niacinamide	Cationic liposomes containing niacinamide	21	28 days	28 days	MI and melasma area were significantly enhanced where cationic liposomes were used, compared with formulations without cationic liposomes, demonstrating a 1.38–2.08-fold improvement.	^214^

## Conclusion and future perspectives

 As previously stated, melasma results from an increase in melanin production by melanocytes near the end of the EP. This layer offer a comprehensive treatment for this condition, but we need more access to them from the standpoint of medications. Energy-based techniques that disrupt the SCboost drug penetration and have adequate efficacy. However, their use has been associated with scarring and surface burns due to post-inflammatory hyperpigmentation, which can be problematic for patients. Dissolvable micro-needles are an efficient and easy-to-use way to treat skin conditions like melasma.

 Furthermore, using micro-needle patches reduces the need for the patient to visit the clinic, and unlike other physical methods, they do not require specialized equipment or employees.

 On the other hand, one of the most recent developments in drug delivery is using nano-drug carriers in skin drug delivery. However, it was found that new generations of nanocarriers, such as NLCs, transferosomes, and noisomes, can have a better effect than conventional nanocarriers because of their flexible structures, which increase their ability to pass through skin pores and improve their ability to reach the end layers of the EP without immediately releasing the drug. Of course, broader and more thorough clinical studies are necessary for a better conclusion.

## Competing Interests

 None declared.

## Ethical Approval

 Not applicable.
